# Heterogeneity in psychological wellbeing within the university community: a latent profile analysis of wellbeing profiles and associated factors

**DOI:** 10.3389/fpsyg.2026.1815021

**Published:** 2026-08-12

**Authors:** Jaume-Miquel March-Amengual, Xavier Sánchez Corrales, Ester Busquets Alibés, Agustí Comella Cayuela

**Affiliations:** 1 Research Group on Methodology, Methods, Models and Outcomes of Health and Social Sciences (M3O), University of Vic-Central University of Catalonia (UVic-UCC), Vic, Spain; 2 Institute for Research and Innovation in Life Sciences and Health in Central Catalonia (IRIS-CC), Vic, Spain; 3 Grifols Foundation Chair of Bioethics, University of Vic-Central University of Catalonia (UVic-UCC), Vic, Spain; 4 Chair of Medical Education, University of Vic-Central University of Catalonia (UVic-UCC), Vic, Spain; 5 Department of Engineering, Faculty of Science, Technology and Engineering, University of Vic-Central University of Catalonia (UVic-UCC), Vic, Spain; 6 Data and Signal Processing Research Group, University of Vic-Central University of Catalonia (UVic-UCC), Vic, Spain; 7 Department of Fundamentals of the Profession and Management in Health, Faculty of Health and Welfare Sciences, University of Vic-Central University of Catalonia (UVic-UCC), Vic, Spain; 8 Department of Psychology, Faculty of Education, Translation, Sport and Psychology, University of Vic-Central University of Catalonia (UVic-UCC), Vic, Spain

**Keywords:** cluster analysis, latent profile analysis, mental health, mental health promotion, psychological wellbeing, social determinants of health, university community

## Abstract

Psychological wellbeing in university settings is a multidimensional construct shaped by individual, occupational, and structural factors that vary across members of the academic community. This study aimed to identify and compare psychological wellbeing profiles among students, Academic and Research Staff (ARS), and Technical, Administrative, and Services Staff (TASS), and to examine the main determinants associated with these profiles. The final sample consisted of 742 participants. In this exploratory study, an unsupervised latent profile analysis was applied to identify wellbeing typologies within each group, and the quality of the solution was evaluated using cohesion, separation, and parsimony indices. In addition, nonparametric tests and logistic regression analysis were conducted to validate profile differentiation and to examine the influence of psychological, sociodemographic, and structural factors on profile membership. The results revealed two distinct profiles of psychological wellbeing (low vs. high) across all three groups, with statistically significant differences across all assessed dimensions (*p* < 0.001) and large effect sizes (Cohen's d ranging from 0.98 to 2.02). Within each group, a higher proportion of students and TASS members were classified in the low wellbeing profile (64.1% and 70.3%, respectively), whereas among ARS the distribution was more balanced (49.7%). These within-group prevalences should not be interpreted as direct between-group comparisons, as profiles were estimated independently within each subpopulation. Logistic regression models demonstrated adequate fit, with pseudo-R^2^ values indicating moderate-to-substantial explanatory strength for profile membership (McFadden R^2^: 20%–32%; Nagelkerke R^2^: 32%–46%). Economic situation emerged as the only significant predictor consistently associated with profile membership across all groups, while variables such as perceived health, sleep quality, psychotropic medication use, and gender showed group-specific effects. These findings suggest the presence of heterogeneity in psychological wellbeing within the university community and highlight the relevance of evidence-informed, differentiated strategies for wellbeing promotion that integrate individual-level interventions with institutional and structural approaches. These findings suggest the presence of heterogeneity in psychological wellbeing within the university community and highlight the relevance of evidence-informed, differentiated strategies for wellbeing promotion that integrate individual-level interventions with institutional and structural approaches. Future longitudinal and multicentre studies are needed to assess the stability and generalizability of these profiles and to inform the development of tailored wellbeing strategies across university subpopulations.

## 1 Introduction

Occupational safety and health has evolved over recent decades from an approach focused almost exclusively on physical hazards toward a more comprehensive perspective that explicitly incorporates psychosocial risks and mental health (Beck and Lenhardt, 2019; Chirico et al., 2019; Kirsten, 2022). This shift has been driven by processes such as digitalization, the intensification of work demands, and the expansion of new work arrangements—particularly remote work—which have been associated with increased stress, mental fatigue, and psychological distress (Lindholm et al., 2024; Moreno, 2022). In our country, psychosocial risks have become one of the leading causes of work-related illness (Departament d'Empresa i Treball, 2021). This situation underscores the need to address physical and psychosocial risks in an integrated manner (World Health Organization, 2022b,c).

Universities represent an organizational context that is particularly vulnerable to psychosocial strain. Structural transformations associated with the adoption of management models inspired by New Public Management, increasing internationalization, growing student numbers, and the intensification of performance evaluation systems have substantially increased both work-related and emotional demands across the academic community (Pereira et al., 2023; Díaz-Patiño and Anaya-Velasco, 2022; Lee and Stensaker, 2021). The COVID-19 pandemic further exacerbated these dynamics, revealing elevated levels of stress, emotional exhaustion, and deterioration in mental health among both university staff and students (Koren et al., 2023; Wray and Kinman, 2021; World Health Organization, 2022a). Empirical evidence and recent review-level synthesis indicate that burnout and related mental health difficulties are prevalent across educational and professional populations, including academic staff, administrative and services staff, and students, with adverse consequences for academic performance, job satisfaction, and wellbeing (Chalghaf et al., 2026; Mudrak et al., 2018; Li et al., 2022; Madigan and Curran, 2021; March-Amengual et al., 2022; Departament de Recerca i Universitats, 2023).

From an organizational and psychological perspective, universities do not constitute neutral environments but rather stratified institutional fields in which wellbeing is unevenly distributed according to individuals' positions (Bourdieu, 1988; Marginson, 2008). Although academic staff, technical and administrative staff, and students share the same institutional context, they are exposed to differentiated configurations of demands, resources, and psychosocial risks (Bruno et al., 2024; Torales et al., 2025).

Among academic staff, the combination of teaching, research, and administrative responsibilities, together with pressure for scientific productivity and employment insecurity, has been associated with higher levels of burnout and lower job satisfaction (Jayman et al., 2022; Cadena-Povea et al., 2025; Wray and Kinman, 2021). TASS, in contrast, have received substantially less attention in published research, despite evidence from several studies indicating that this group exhibits elevated levels of emotional exhaustion, organizational cynicism, and technostress-related anxiety (Akbas et al., 2018; Guinez-Perez et al., 2022; Díaz-Patiño and Anaya-Velasco, 2022). Students face high academic demands, performance pressure, and employment uncertainty, which have been consistently linked to anxiety, depression, and emotional exhaustion (Schaufeli et al., 2002; Li et al., 2022; Departament de Recerca i Universitats, 2023).

Previous studies highlights the importance of addressing psychosocial risks through evidence-based interventions and emotional skills training; however, research on the effectiveness of specific programs in higher education remains limited (Corpuz, 2023; Jayman et al., 2022; Wray and Kinman (2021); Departament de Recerca i Universitats, 2023).

Within this context, exploratory person-centered approaches are needed to identify distinct psychological wellbeing profiles and their sociodemographic and health-related correlates. Previous studies of mixed university populations have compared mental health outcomes between students and university staff but have generally relied on variable-centered analyses and often grouped academic and non-academic employees together (Odriozola-González et al., 2020; Meeks et al., 2023). Conversely, person-centered research has focused predominantly on student-only samples, demonstrating that positive wellbeing and psychological distress can combine into distinct profiles (Guelmami et al., 2023; Hernández-Torrano and Ibrayeva, 2025). Thus, joint person-centered profiling of students, academic staff, and technical and administrative staff remains largely unexplored. The present study addresses this gap by identifying shared wellbeing profiles across the university community and examining their sociodemographic, health-related, and institutional correlates.

## 2 Methodology

### 2.1 Study design

An observational study with a cross-sectional design was conducted to estimate prevalence rates and examine associations between variables. The sample was recruited through a voluntary participation procedure following an open invitation sent via institutional email to students, ARS and TASS between March and May 2025. In line with this recruitment strategy, the sample should be classified as non-probabilistic due to self-selection.

The psychological wellbeing assessment approach was designed to minimize stigmatization and avoid perceptions of institutional surveillance. The study was presented from a non-clinical and salutogenic perspective (“How are you feeling today?”), emphasizing the individual contribution to collective wellbeing. In the case of ARS and TASS, dissemination was carried out through prevention delegates (worker representatives on the health and safety committee), fostering a climate of trust, transparency, and participation.

Prior to the final administration of the questionnaire, a pilot study was conducted with 63 participants (21 Students, 22 ARS, and 20 TASS). For students, the questionnaire was administered in person to collect qualitative feedback on item wording and clarity. For ARS and TASS, the pilot survey was distributed via email with the same objective. As a result of this process, several open-ended items were revised. Data from the pilot study were not included in the final sample.

The following variables (Table 1) were collected for the three groups: gender, age, nationality, self-perceived health status, economic situation, length of time at the university, faculty or workplace, degrees pursued or taught, and employment status. In addition, health-related variables were recorded, including perceived health, smoking status, alcohol and drug use, sleep duration and quality, and the use of anxiolytic, antidepressant, or hypnotic/sedative medications.

**Table 1:** Variables, types, and categories.

Variable	Type	Categories
Gender	Binary	2
Age	Ordinal	3
Nationality	Binary	2
Self-perceived health	Ordinal	5
Economic situation	Ordinal	4
Sports practice	Binary	2
Physical activity hours	Ordinal	6
Alcohol risk	Binary	2
Tranquilizers/Hypnotics	Binary	2
Drug use	Binary	2
Smoker	Nominal	3
Cigarettes per day	Ordinal	4
Sleep quality	Binary	2
Studies	Nominal	5
Course	Ordinal	7
Wellbeing cluster	Binary	2

### 2.2 Participants and procedure

The target population comprised the 10,887 members of the university community corresponding to the 2024–2025 academic year at the Universitat de Vic–Universitat Central de Catalunya (UVic–UCC), located in Catalonia (Spain). This population included students enrolled in undergraduate, master's, and doctoral programs at the UVic and Elisava campuses, as well as ARS and TASS (Universitat de Vic-Universitat Central de Catalunya, 2025). A personalized email invitation was distributed between March and May 2025, accompanied by two reminders (at weeks 3 and 7). The invitation included a link to a self-administered questionnaire hosted on the REDCap platform; participation was entirely voluntary and no incentives were offered.

A total of 742 participants (Table 2) completed the survey out of 10,887 invited individuals, distributed across 368 students (4.2%), 189 ARS (12.9%), and 185 TASS (31.6%).

**Table 2:** Descriptive main characteristics of the overall sample.

Variable	Category	Overall sample (*N* = 742)	Students (*n* = 368)	ARS (*n* = 189)	TASS (*n* = 185)
Gender	Men	234 (31.5)	112 (30.4)	74 (39.2)	48 (25.9)
	Women	508 (68.5)	256 (69.6)	115 (60.8)	137 (74.1)
Age group (years)	19–29	369 (50.1)	344 (93.5)	4 (2.2)	21 (11.5)
	30–39	81 (11.0)	12 (3.3)	35 (18.8)	34 (18.6)
	40–49	134 (18.2)	5 (1.4)	75 (40.3)	54 (29.5)
	50–59	114 (15.5)	6 (1.6)	49 (26.3)	59 (32.2)
	60–69	39 (5.3)	1 (0.3)	23 (12.4)	15 (8.2)
Self-rated health	Very poor	5 (0.7)	3 (0.8)	0 (0.0)	2 (1.1)
	Poor	30 (4.0)	15 (4.1)	6 (3.2)	9 (4.9)
	Neither good nor poor	124 (16.7)	68 (18.5)	26 (13.8)	30 (16.2)
	Good	434 (58.5)	209 (56.8)	117 (61.9)	108 (58.4)
	Very good	149 (20.1)	73 (19.8)	40 (21.2)	36 (19.5)
Use of tranquilizers/sleeping pills	Yes	151 (20.5)	79 (21.6)	34 (18.4)	38 (20.7)
	No	584 (79.5)	287 (78.4)	151 (81.6)	146 (79.3)
Perceived economic situation	Precarious	70 (9.4)	41 (11.1)	12 (6.3)	17 (9.2)
	Average	343 (46.2)	165 (44.8)	69 (36.5)	109 (58.9)
	Good	299 (40.3)	147 (39.9)	96 (50.8)	56 (30.3)
	Very good	30 (4.0)	15 (4.1)	12 (6.3)	3 (1.6)
Gets enough rest from sleep	Yes	372 (50.6)	153 (41.8)	107 (57.8)	112 (60.9)
	No	363 (49.4)	213 (58.2)	78 (42.2)	72 (39.1)

Values are reported as *n* (%), with percentages calculated on the basis of valid responses within each column. The overall sample included students, ARS and TASS participants. Valid *n* differed slightly across variables due to missing data: age group, overall *n* = 737, students *n* = 368, ARS *n* = 186, TASS *n* = 183; use of tranquilizers/sleeping pills, overall *n* = 735, students *n* = 366, ARS *n* = 185, TASS *n* = 184; gets enough rest from sleep, overall *n* = 735, students *n* = 366, ARS *n* = 185, TASS *n* = 184. Gender, self-rated health, and perceived economic situation had complete data for the columns shown.

The overall response rate was 6.8%, which is lower than the response rates typically reported in institutional web-based health surveys in university populations [e.g., American College Health Association (NCHA) institutional averages: 10%–12%; Baruch and Holtom, 2008]. This relatively low participation rate suggests a potential risk of non-response bias, which could not be formally assessed in the present study. In particular, the lower response rate observed among students (4.2%) indicates that these estimates may not fully represent the general student population and should therefore be interpreted with caution.

A marked variability in participation was observed across groups, with a lower response rate among students (4.2%), an intermediate rate among ARS (12.9%), and a higher rate among TASS (31.6%). This heterogeneity in participation rates further reinforces concerns regarding subgroup representativeness and highlights the likelihood of non-response bias, particularly among students.

### 2.3 Inclusion and exclusion criteria

Students enrolled in official academic programs, as well as ARS and TASS members with an active employment contract, were included in the study. All participants provided informed consent. Individuals with language difficulties that could hinder adequate comprehension of the questionnaire (particularly international students with limited language proficiency) were excluded, as were incomplete questionnaires.

**Figure 1:**
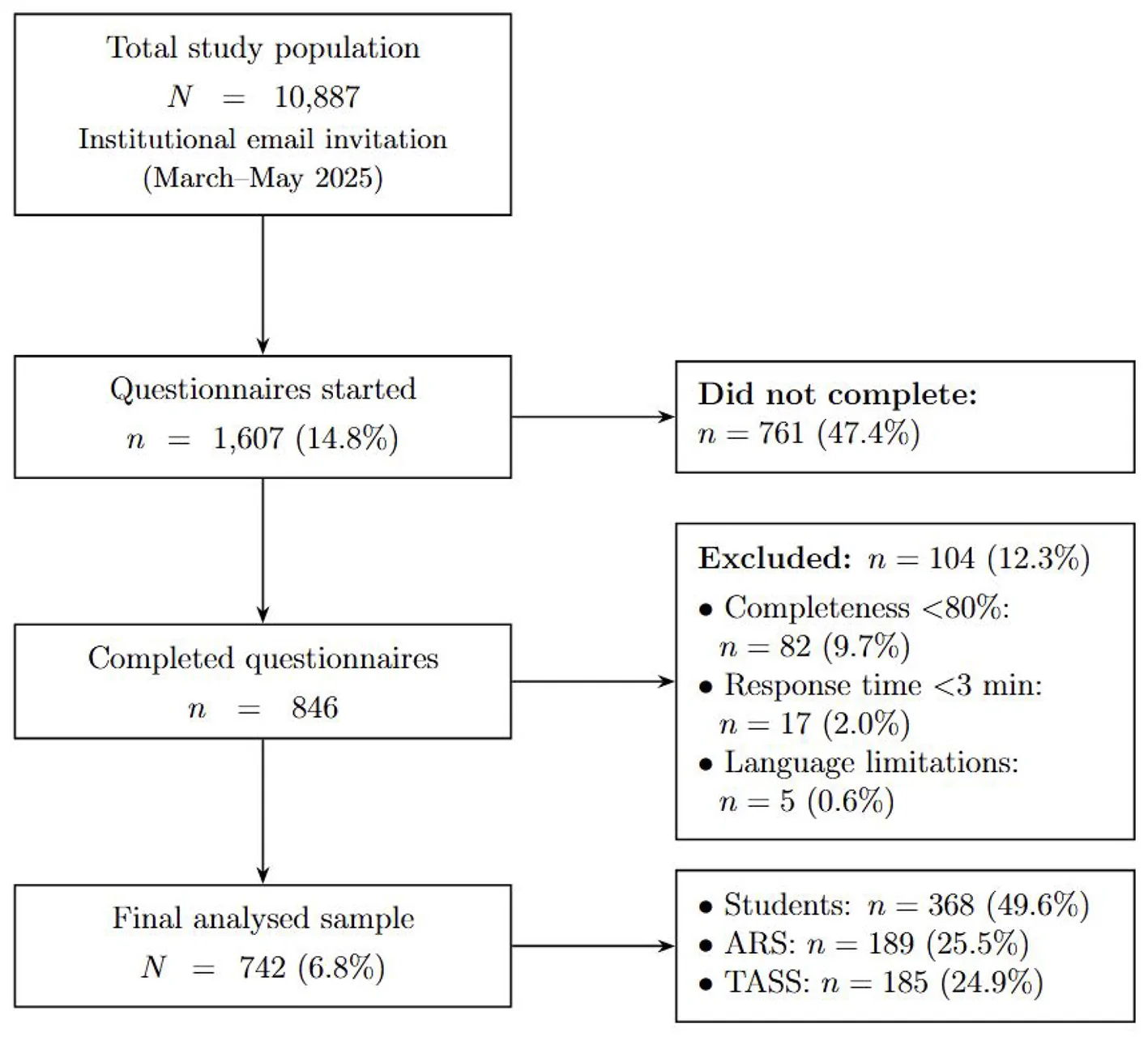
Flow diagram of participants.

The following flow diagram of participants (Figure 1) summarizes the process of participant identification and selection in this study.

Questionnaires with missing values in any item of scale or sub-scale were excluded using listwise deletion. This procedure was adopted as a conservative approach because the missing-data mechanism could not be verified. Applying imputation methods would have required assuming that missing values were Missing At Random (MAR) or Missing Completely At Random (MCAR), assumptions that could not be guaranteed in the present study. This measure was adopted because, given the brevity of some scales and subscales, the absence of a single item compromises between 17% and 25% of the dimensional information, making any missing value estimation particularly sensitive (Hair et al., 1999; Little and Rubin, 2002).

### 2.4 Instruments

Psychological distress was assessed using the *Brief Symptom Inventory 18* (BSI-18), a brief self-administered inventory validated in Spanish that measures emotional distress (Derogatis, 2013; Andreu et al., 2008). The instrument comprises 18 items distributed across three subscales, somatization (6 items), anxiety (6 items), and depression (6 items) rated on a Likert scale ranging from 0 to 4. In addition, it provides a global indicator of emotional distress, the Global Severity Index (GSI).

In the present study, the internal consistency of the instrument was assessed using Cronbach's alpha coefficient, which yielded a value of 0.924. The alpha based on standardized items was 0.925, confirming the stability of the scores regardless of the measurement scale.

To assess burnout, the Spanish-validated versions of the Maslach Burnout Inventory were used: the Maslach Burnout Inventory–Student Survey (MBI-SS; Schaufeli et al., 2002) for students, employing the Spanish adaptation by Hederich-Martínez and Caballero-Domínguez, 2016 and the Maslach Burnout Inventory–General Survey (MBI-GS) for staff, using validated Spanish versions (Salanova et al., 1999; Gil-Monte, 2002; Guevara Bedoya and Ocampo Agudelo, 2014; De Beer et al., 2024). Both instruments assess three core dimensions of burnout: emotional exhaustion (five items), cynicism (four items), and personal accomplishment professional efficacy or academic efficacy (six items), yielding a total of 15 items in each version. Responses were recorded on a Likert scale ranging from 0 (never) to 6 (every day). The MBI does not provide a global score; therefore, each dimension was analyzed separately. In the present study, burnout-related dimensions were treated as continuous indicators: emotional exhaustion reflects feelings of being emotionally overextended and depleted by work or study demands; cynicism reflects psychological detachment from work or study and from others; and personal accomplishment reflects perceived competence and achievement. No diagnostic cut-off points were applied, and the study did not classify participants as having or not having burnout. The MBI-GS and MBI-SS assess three conceptually comparable dimensions—exhaustion, cynicism, and professional or academic efficacy—adapted to occupational and academic contexts, respectively (Schaufeli et al., 2002; Hederich-Martínez and Caballero-Domínguez, 2016). Accordingly, comparisons were made at the conceptual dimension level.

In this study, the MBI-SS showed Cronbach's α = 0.714 and standardized α = 0.691, indicating acceptable reliability for exploratory research. For the MBI-GS, α = 0.798 and standardized α = 0.761, considered adequate according to standard social science criteria.

Lastly, life satisfaction was assessed using the Satisfaction With Life Scale (SWLS; Diener et al., 1985). The Spanish version of the SWLS consists of five items rated on a 7-point Likert scale (1 = strongly disagree; 7 = strongly agree). This adaptation has shown adequate convergent validity, correlating positively with indicators of psychological wellbeing and negatively with measures of distress (Vázquez et al., 2013), and has been validated in various populations, including the general population, older adults, and university students. In the present study, the scale demonstrated reliability with a Cronbach's alpha of 0.889 and an alpha based on standardized items of 0.897.

Global psychological wellbeing was operationalized as an integrative composite index combining indicators of psychological distress, emotional energy, reversed cynicism, personal accomplishment, and life satisfaction. This operationalization was theoretically informed by the Job Demands–Resources model (JD-R; Bakker and Demerouti, 2017), in which wellbeing can be understood as the balance between accumulated psychological demands and available personal resources. Accordingly, the index was not intended to represent a definitive reflective higher-order latent construct, but rather an operational approximation of the individual's overall psychological state.

To operationalize this index, five indicators were derived from the three validated instruments described above: (1) the T-score of the Global Severity Index (GSI) from the BSI-18 (18 items), a standardized score indicating the level of psychological distress relative to a reference population; (2) the total score of the Emotional Exhaustion subscale of the MBI (5 items); (3) the total score of the Cynicism subscale of the MBI (4 items); (4) the total score of the Personal Accomplishment subscale of the MBI (6 items); and (5) the total score of the Satisfaction With Life Scale (SWLS; 5 items).

To ensure metric comparability across indicators derived from instruments with different ranges and numbers of items, all scores were rescaled to a common 0–100 scale using a linear min-max transformation shown in (Equation 1):


$$\begin{array}{c} X_{\text{rescaled}}=\frac{X-X_{\min}}{X_{\max}-X_{\min}}\times 100 \end{array}$$ (1)

where *X* represents the original score and *X*_min_ and *X*_max_ correspond to the theoretical minimum and maximum values of each variable.

As a linear transformation, this operation preserves both the ordinal relationships between scores and the correlational structure of the original variables. Unlike standardization via *z*-scores, rescaling to a bounded range prevents variables with greater variance or scale amplitude from exerting a disproportionate influence on distance calculations, while maintaining the substantive interpretability of the resulting profiles (Mooi and Sarstedt, 2011).

This approach is particularly appropriate when combining heterogeneous measures, as it homogenizes the metric without eliminating the structural information of each variable (Hair et al., 2019). Consequently, the rescaling applied balances the contribution of variables in the clustering process and facilitates a comparative interpretation of cluster profiles (Table 3).

**Table 3:** Theoretical minimum and maximum values used to rescale the wellbeing variables.

Variable	Min	Max
GSI (males)	37	79
GSI (females)	35	81
EE	0	30
CY	0	24
PA	0	36
SWLS	5	35

GSI, Global Severity Index; EE, Emotional Exhaustion; CY, Cynicism; PA, Personal Accomplishment; SWLS, Satisfaction With Life Scale.

Subsequently, the following rescaled variables were inverted so that higher scores reflect greater wellbeing (100−*X*):

- The BSI-18 T-score (GSI) was inverted to obtain the Reversed Global Severity Index (R-GSI), indicating psychological wellbeing, such that higher values reflect lower psychological distress.
- The MBI Emotional Exhaustion (EE) subscale was transformed into Emotional Energy (EEn), where higher values indicate lower emotional exhaustion, that is, greater vitality and coping capacity in response to academic or occupational demands.
- The MBI Cynicism subscale (CY) was transformed into Reversed Cynicism (R-CY), where higher values indicate lower cynicism, that is, greater emotional connection with work or studies.

The global psychological wellbeing construct was thus defined by the three inverted variables (R-GSI, EEn, R-CY) together with Personal Accomplishment (PA) from the MBI and the Life Satisfaction score (LS) from the SWLS.

### 2.5 Data analysis

Data analysis was conducted using SPSS v31, FACTOR (version 12.06.08) and the VSCode programming environment (version 1.106.2) with Python (version 3.11.9). Descriptive analyses began with correlations between sociodemographic and health habit variables to identify any significant relationships using the appropriate statistics according to variable type.

**Figure 2:**
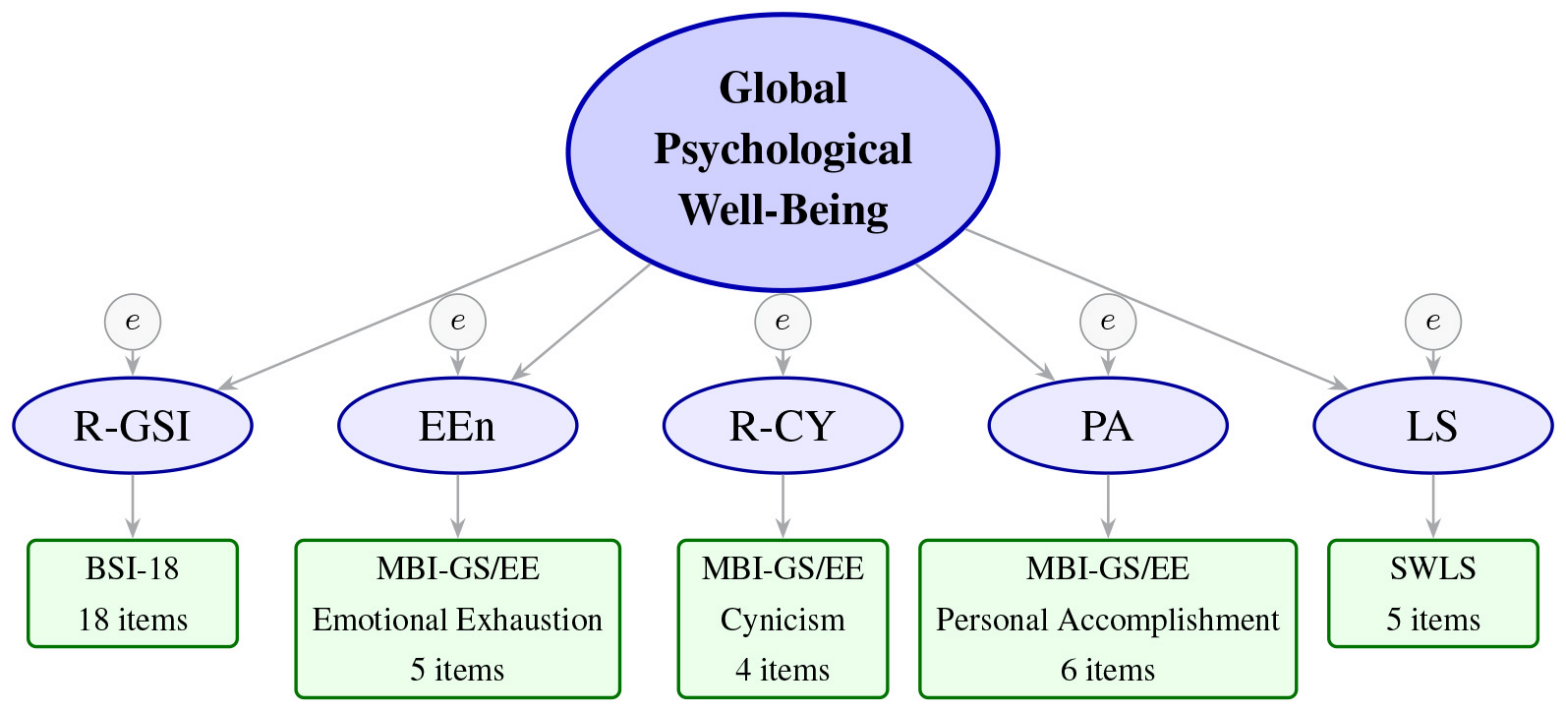
Conceptual operational model used to construct the global psychological wellbeing index. The figure represents the conceptual organization of the five indicators used to operationalize global psychological wellbeing. Exploratory diagnostics supported examination of a common latent structure among the five indicators ($\bar{r}=0.42$, KMO = 0.75, Bartlett's test *p* < 0.001); CFA results are reported in Table 5.

The five indicators (R-GSI, EEn, R-CY, PA, and LS) were examined to assess whether they showed sufficient empirical coherence to justify their joint use in an operational index of global psychological wellbeing. Exploratory and confirmatory factor analytic procedures were used as complementary checks of this coherence, rather than as definitive validation of a reflective latent measurement model. The indicators were then incorporated into the subsequent Latent Profile Analysis (LPA) to identify within-group wellbeing profiles. Figure 2 presents the conceptual organization of the indicators used to operationalize the global psychological wellbeing index.

To examine the empirical coherence of the five indicators included in the operational index of global psychological wellbeing, an exploratory factor analysis (EFA) was conducted on R-GSI, EEn, R-CY, PA, and LS with 742 participants, using a Pearson correlation matrix and the Robust Unweighted Least Squares (RULS) extraction method implemented in the FACTOR software (Table 4).

**Table 4:** Pearson correlation matrix of the five wellbeing variables (*N* = 742).

Variable	R-GSI	EEn	R-CY	PA	LS
R-GSI	—	0.655	0.451	0.341	0.504
EEn		—	0.543	0.288	0.371
R-CY			—	0.338	0.313
PA				—	0.439
LS					—

GSI, Global Severity Index; EEn, Emotional Energy; R-CY, Reversed Cynicism; PA, Personal Accomplishment; LS, Life Satisfaction.

Nine of the 10 off-diagonal correlations exceeded the conventional inclusion threshold of 0.30 (Hair et al., 2019), with the only exception being the correlation between emotional energy and personal accomplishment (*r* = 0.288), which fell marginally below this criterion. The mean inter-item correlation of 0.424 reflects a moderate-to-high degree of shared variance, consistent with the presence of a common latent factor. The strongest association was observed between R-GSI and EEn (*r* = 0.655), indicating a particularly close relationship between these two indicators. Correlations involving EEn and PA were at the lower end of the range, although they remained positive and substantively meaningful.

Overall, the pattern of positive and relatively homogeneous intercorrelations suggests an empirical basis for considering the five scores jointly as indicators of an operational global psychological wellbeing index.

The correlation matrix was adequate for factor analysis (*KMO* = 0.75, 95% CI [0.71, 0.79]; Bartlett's test of sphericity, χ^2^(10) = 1, 119.60, *p* < 0.001), and optimal parallel analysis indicated the adequacy of a unidimensional structure. The single extracted factor explained 54.32% of the total variance (eigenvalue = 2.72), with moderate-to-high factor loadings (λ = 0.49–0.80) whose 95% BCa confidence intervals excluded trivial values, and communalities ranging from 0.24 to 0.64. Factor reliability was adequate (ω = 0.82) and factor determinacy was high (*FDI* = 0.91), supporting the use of the indicators as a parsimonious operational approximation for the subsequent exploratory profile analysis.

To examine the latent structure of the five standardized indicators, two alternative confirmatory models were compared (Table 5) using Mild-Restricted Confirmatory Factor Analysis (Lorenzo-Seva, 2026), based on a Pearson correlation matrix, the LOSEFER empirical correction for scaling the chi-square statistic, and BCa bootstrap confidence intervals derived from 500 samples. The one-factor model showed a mixed fit pattern: *RMSEA* = 0.140, 95% CI [0.105, 0.182]; *CFI* = 0.949, 95% CI [0.903, 0.974]; *NNFI* = 0.897, 95% CI [0.805, 0.947]; *GFI* = 0.962; *AGFI* = 0.925; and *RMSR* = 0.084, 95% CI [0.059, 0.110], exceeding the Kelley criterion (0.037). These results indicate that the fit of the one-factor model was not unequivocally satisfactory, particularly regarding RMSEA and RMSR.

**Table 5:** Goodness-of-fit indices and factor loadings for the one-factor and two-factor CFA models (*N* = 742).

	One-factor model	Two-factor model
Model specification		
Approach	Mild-Restricted CFA	
Degrees of freedom	5	4
Goodness-of-fit indices		
RMSEA [95% CI]	0.140 [0.105, 0.182]^*a*^	0.062 [0.041, 0.123]^*b*^
CFI [95% CI]	0.949 [0.903, 0.974]^*b*^	0.992 [0.970, 0.997]^*c*^
NNFI [95% CI]	0.897 [0.805, 0.947]^*a*^	0.980 [0.926, 0.993]^*c*^
GFI [95% CI]	0.962 [0.938, 0.978]^*c*^	0.994 [0.976, 0.997]^*c*^
AGFI [95% CI]	0.925 [0.876, 0.957]^*b*^	0.985 [0.941, 0.994]^*c*^
RMSR [95% CI]	0.084 [0.059, 0.110]	0.152 [0.093, 0.414]
BIC	143.70	114.53
Largest standardized residual	4.47	7.69
Factor loadings λ [95% CI]		
*F1*		
R-GSI	0.724 [0.653, 0.786]	0.533 [0.277, 0.778]
EEn	0.678 [0.609, 0.743]	0.977 [0.842, 1.064]^†^
R-CY	0.648 [0.589, 0.699]	0.447 [0.175, 0.654]
PA	0.521 [0.431, 0.590]	—
LS	0.626 [0.552, 0.676]	—
*F2*		
PA	—	0.765 [0.401, 4.728]^‡^
LS	—	0.681 [0.536, 0.844]
*Inter-factor correlation and reliability*		
*r* F1–F2 [95% CI]	—	0.611 [0.560, 0.707]
EAP reliability/ORION	0.787	F1: 0.956 F2: 0.739
Factor Determinacy Index	0.887	F1: 0.978 F2: 0.859

CFA, Confirmatory Factor Analysis. Confidence intervals (95%) were obtained using bias-corrected and accelerated bootstrap (500 samples). RMSEA, Root Mean Square Error of Approximation; CFI, Comparative Fit Index; NNFI, Non-Normed Fit Index; GFI, Goodness-of-Fit Index; AGFI, Adjusted Goodness-of-Fit Index; RMSR, Root Mean Square of Residuals (Kelley criterion = 0.037); BIC, Bayesian Information Criterion (lower values indicate better fit). EAP reliability/ORION refers to the reliability of factor score estimates obtained using the ORION/ORIONz procedure for Expected A Posteriori (EAP) factor score estimation. FDI = Factor Determinacy Index. Fit evaluation: ^*a*^ poor; ^*b*^ fair; ^*c*^ excellent (thresholds: RMSEA >0.10 poor, 0.05–0.10 fair; CFI/NNFI ≥0.95 excellent, 0.92–0.95 fair). ^†^ Heywood case: loading exceeds 1.0, indicating estimation instability. ^‡^ Anomalously wide confidence interval indicating weak factor identification.

However, the elevated RMSEA should be interpreted with caution because this index may overestimate model misfit in models with few degrees of freedom and large samples. In addition, despite the residual misfit suggested by RMSR, all factor loadings were positive and statistically significant (λ = 0.521–0.724), with bootstrap confidence intervals excluding zero, and factor score reliability was acceptable, as indicated by Expected A Posteriori reliability (*EAP* = 0.787) and the Factor Determinacy Index (*FDI* = 0.887). Therefore, the one-factor model was considered a parsimonious empirical approximation supporting the operational index of global psychological wellbeing for the purposes of subsequent exploratory profile analysis, rather than a definitive reflective measurement model.

The two-factor model yielded better global fit indices: *RMSEA* = 0.062, 95% CI [0.041, 0.123]; *CFI* = 0.992, 95% CI [0.970, 0.997]; *NNFI* = 0.980, 95% CI [0.926, 0.993]; *GFI* = 0.994; and *AGFI* = 0.985. However, this apparently better fit was accompanied by relevant psychometric instability, including an excessively high loading for Emotional Exhaustion (λ = 0.977, 95% CI [0.842, 1.064]), consistent with a possible Heywood case, an extremely wide confidence interval for Personal Accomplishment (95% CI [0.401, 4.728]), and abnormally large standardized residuals (max. = 7.69).

Thus, although the two-factor model improved some global fit indices, its parameter instability and residual misfit undermined its interpretability and psychometric reliability. For this reason, the one-factor solution was retained as a parsimonious and psychometrically stable empirical approximation supporting the operational index of global psychological wellbeing for the purposes of the subsequent exploratory profile analyses, rather than as a definitive reflective measurement model. This decision should be understood as a pragmatic operational choice for profile construction, pending replication and further testing with alternative measurement specifications, including bifactor, hierarchical, and composite/formative models, in independent samples. Because this operational index underlies the subsequent profile analyses, the resulting wellbeing profiles should be interpreted as conditional on an imperfectly fitting one-factor structure. It should be noted, however, that the Latent Profile Analysis itself was conducted directly on the five continuous wellbeing indicators as separate inputs to the mixture model, rather than on a single reduced factor score derived from the one-factor CFA; the clustering solution is therefore not mechanically dependent on the fit of the measurement model, although its conceptual justification remains tied to it.

Future studies should replicate these findings in independent samples and examine bifactor or hierarchical models that allow for a more comprehensive representation of the latent structure of the construct.

Next, statistical associations between variables were assessed, considering (*p* < 0.05) from the χ^2^ test as the threshold for statistical significance, and Cramer's V was used to measure the strength of the associations.

Finally, a pattern analysis of these variables within the clusters was conducted, considering the percentage distribution in both clusters, the identification of discriminative categories (with a relevance threshold above 20%), and statistical significance using Pearson's χ^2^ test (*p* < 0.05).

The analytical approach followed four sequential steps applied independently to each group. First, Latent Profile Analysis (LPA) was used to identify wellbeing profiles, with the optimal number of profiles selected based on multiple criteria. Second, profiles were characterized by comparing the five wellbeing indicators across clusters using the Mann–Whitney *U* test and Cohen's *d* (95% CI via bootstrapping), complemented by a profile plot displaying cluster and group means. Third, a descriptive radar chart summarizing the relative contribution of each wellbeing indicator to cluster differentiation within each group (based on mean absolute SHAP values per cluster, derived from the Random Forest model) is presented as an exploratory internal visualization. Fourth, sociodemographic and health-related variables (Table 1) were modeled as correlates of profile membership using multivariate logistic regression (Firth penalized regression for TASS).

Throughout this article, the term Latent Profile Analysis (LPA) is used to refer to the finite mixture modeling procedure applied to identify latent subgroups within the data. The continuous wellbeing variables were modeled using the Python package StepMix, which internally implements a Gaussian mixture model, consistent with the formal requirements of LPA. The choice of LPA was informed by previous student mental health research using person-centered latent profile models to identify heterogeneous patterns combining psychological symptoms and positive wellbeing indicators (Guelmami et al., 2023).

Through this exploration, two clusters were identified for each group, resulting in a dichotomous variable, Wellbeing cluster: 0 (individuals with lower wellbeing) and 1 (higher wellbeing).

An independent clustering analysis was performed for each population group. Group-specific models allow capturing the particular wellbeing dynamics of each university subpopulation, providing greater interpretability and theoretical coherence with the differential demands that characterize each group. Clustering was conducted using the five previously described wellbeing variables (R-GSI, EEn, R-CY, PA, and LS).

Group-specific clustering models were preferred over a single joint model with group as a covariate, for both theoretical and methodological reasons. Theoretically, Students, ARS, and TASS represent sub-populations with structurally distinct roles, contractual arrangements, and psychosocial demands, for which an equivalent wellbeing structure cannot be assumed a priori (Schaufeli et al., 2002; Mudrak et al., 2018; Bruno et al., 2024). Methodologically, joint models (including multigroup LPA) require prior evidence of at least configural measurement invariance across groups, a condition that cannot be presumed given the substantive differences between groups. Group-specific models therefore represent the most conservative and theoretically coherent option for the diagnostic objectives of this study.

The cluster analysis separated the population into two coherent groups using the five variables, indicating that these variables capture a common underlying dimension—psychological wellbeing—which is the construct that effectively organizes individuals into distinct profiles, as confirmed by the factor analysis.

To examine variable importance in differentiating wellbeing clusters, a Random Forest model with 50 decision trees was trained using the five global psychological wellbeing factor variables (R-GSI, EEn, R-CY, PA, and LS) as predictors and cluster membership as the target variable. The model automatically computes the importance of each variable using the Mean Decrease in Impurity, which quantifies how much each variable contributes to improving split purity in the trees.

From the trained model, two complementary visualizations were generated: a SHAP (SHapley Additive exPlanations) plot showing the individual contribution of each variable to each participant's cluster classification, where each point represents a participant and color indicates whether the variable has high (blue) or low (red) values; and a radar chart representing the mean absolute SHAP value of each variable per cluster, allowing visual comparison of the relative contribution of each indicator to the classification of participants within each wellbeing profile.

Subsequently, a multivariate logistic regression analysis was conducted to predict cluster membership for each of the three groups based on the following variables: Perceived Health, Economic Situation, use of Tranquilizers/Hypnotics, Sleep Quality, Drug Consumption, Sports Practice, Hours of Physical Activity, and Gender. For this analysis, categorical variables were first transformed into binary indicators (0/1) by dichotomizing each category using the get_dummies method from the Python *pandas* library (parameterized as drop_first=True to avoid perfect multicollinearity, also known as the “dummy variable trap”). All binary predictors were coded as 1 (presence) and 0 (absence, reference category). Specifically: Gender (1 = female, 0 = male), Tranquilizers/Hypnotics (1 = uses, 0 = does not use), Drug Consumption (1 = uses, 0 = does not use), and Sports Practice (1 = practices, 0 = does not). Two distinct sleep-related variables were included: Sleep Quality (1 = good sleep quality, 0 = poor sleep quality) and Rest (1 = adequate rest, 0 = inadequate rest). Self-perceived health (1 = very poor to 5 = very good), Economic Situation (1 = very precarious to 4 = very good), and Hours of Physical Activity were entered as ordinal predictors. The outcome variable was coded 1 for membership in the low wellbeing cluster (Cluster 0) and 0 for membership in the high wellbeing cluster (Cluster 1), consistent with the cluster coding introduced in Section 3.1 and used throughout Section 3.2. An intercept (β_0_) was included to prevent the model from forcing probabilities to 0.5 when all predictors are zero. Finally, algorithmic optimization was applied by maximizing the log-likelihood to estimate the β coefficients that best predict cluster membership using the Maximum Likelihood Estimation (MLE) method.

Economic situation (four ordered categories, 1–4) and self-perceived health (five ordered categories, 1–5) were entered as continuous linear terms, assuming equal spacing and a constant log-odds effect across adjacent categories. It should be noted that the sample sizes used in the logistic regression analysis differ slightly from those employed in the latent profile analysis due to listwise deletion of cases with missing values in one or more sociodemographic predictor variables. The final sample sizes for the logistic regression were: Students (*n* = 365), ARS (*n* = 182), and TASS (*n* = 180), compared to *n* = 368, 189, and 185 in the profile analysis.

### 2.6 Ethical considerations

The study was conducted in accordance with the Declaration of Helsinki and applicable regulations on research involving human participants. Ethical approval was granted by the Research Ethics Committee of the Universitat de Vic–Universitat Central de Catalunya (Approval No. 329/2024). Participation was voluntary, and electronic informed consent was obtained from all participants, who were informed of their right to withdraw from the study. Anonymity was ensured throughout the research process. Data were stored on secure institutional servers in compliance with Regulation (EU) 2016/679 (General Data Protection Regulation, GDPR) and Spanish Organic Law 3/2018 on the Protection of Personal Data and Guarantee of Digital Rights.

## 3 Results

The following section presents the results obtained for the main variables included in the analysis.

Table 6 presents the descriptive statistics of the variables in their original scale, whereas Table 7 reports the same dimensions after rescaling to a common scale of 0 to 100 and the inverted indicators (R-GSI, EEn, and R-CY), where higher values indicate more favorable psychological functioning. This procedure facilitates comparability across dimensions and enables their use in subsequent multivariate analyses, including factor and cluster analyses.

**Table 6:** Descriptive statistics of the original instrument dimensions across the total sample and groups.

Dimension	Total (*N* = 742)	Students (*n* = 368)	ARS (*n* = 189)	TASS (*n* = 185)
Psychological distress (GSI)	56.89 [56.17, 57.62] (10.06)	60.89 [60.01, 61.76] (8.52)	52.34 [50.91, 53.78] (9.98)	53.59 [52.17, 55.02] (9.85)
Emotional Exhaustion (EE)	2.95 [2.83, 3.07] (1.69)	3.56 [3.40, 3.72] (1.58)	2.40 [2.17, 2.62] (1.58)	2.30 [2.07, 2.53] (1.58)
Cynicism (CY)	1.69 [1.58, 1.81] (1.59)	1.81 [1.65, 1.98] (1.62)	1.49 [1.28, 1.71] (1.52)	1.66 [1.43, 1.89] (1.58)
Personal accomplishment (PA)	4.61 [4.53, 4.69] (1.09)	4.19 [4.08, 4.31] (1.12)	5.06 [4.95, 5.18] (0.78)	4.97 [4.83, 5.11] (0.99)
Life satisfaction (LS)	23.48 [23.00, 23.95] (6.61)	22.39 [21.70, 23.08] (6.71)	25.75 [24.91, 26.58] (5.83)	23.32 [22.37, 24.28] (6.61)

Values are reported as mean [95% confidence interval] (standard deviation).

**Table 7:** Descriptive statistics of the rescaled study dimensions (0–100) across the total sample and groups.

Dimension (0–100)	Total (*N* = 742)	Students (*n* = 368)	ARS (*n* = 189)	TASS (*n* = 185)
Psychological distress inv. (R-GSI)	52.45 [50.82, 54.08] (22.58)	43.51 [41.55, 45.48] (19.13)	62.63 [59.41, 65.86] (22.47)	59.82 [56.62, 63.02] (22.09)
Emotional Energy (EEn)	50.86 [48.82, 52.89] (28.21)	40.70 [37.99, 43.40] (26.38)	60.07 [56.28, 63.86] (26.41)	61.66 [57.84, 65.48] (26.33)
Reversed Cynicism (R-CY)	71.79 [69.89, 73.70] (26.46)	69.79 [67.03, 72.56] (26.97)	75.11 [71.48, 78.74] (25.27)	72.39 [68.56, 76.22] (26.39)
Personal accomplishment (PA)	76.79 [75.48, 78.10] (18.19)	69.84 [67.93, 71.76] (18.69)	84.39 [82.53, 86.25] (12.97)	82.85 [80.46, 85.24] (16.46)
Life satisfaction (LS)	61.59 [60.01, 63.18] (22.03)	57.97 [55.68, 60.26] (22.37)	69.15 [66.36, 71.94] (19.44)	61.08 [57.89, 64.28] (22.03)

Values are reported as mean [95% confidence interval] (standard deviation).

Overall, the descriptive results based on the variables studied suggested moderate mean scores for psychological distress and burnout-related dimensions in the total sample, together with relatively high scores for personal accomplishment and life satisfaction. However, analyses stratified by group revealed a consistent pattern of differences.

Specifically, the student group exhibited less favorable scores across most indicators, characterized by higher levels of psychological distress, emotional exhaustion, and cynicism, as well as lower levels of personal accomplishment and life satisfaction. In contrast, university staff groups (TASS and ARS) showed more adaptive profiles, with lower levels of distress and burnout, and higher values in positive wellbeing indicators. These differences were consistently observed both in the original variables and in the rescaled variables (3 inverted), reinforcing the robustness of the findings and ruling out the possibility that the observed pattern was attributable to differences in the metrics of the scales used.

For the cluster analysis, the absence of multicollinearity was verified both in the overall sample and separately within the three analysis groups.

### 3.1 Latent profile analysis of wellbeing

**Figure 3:**
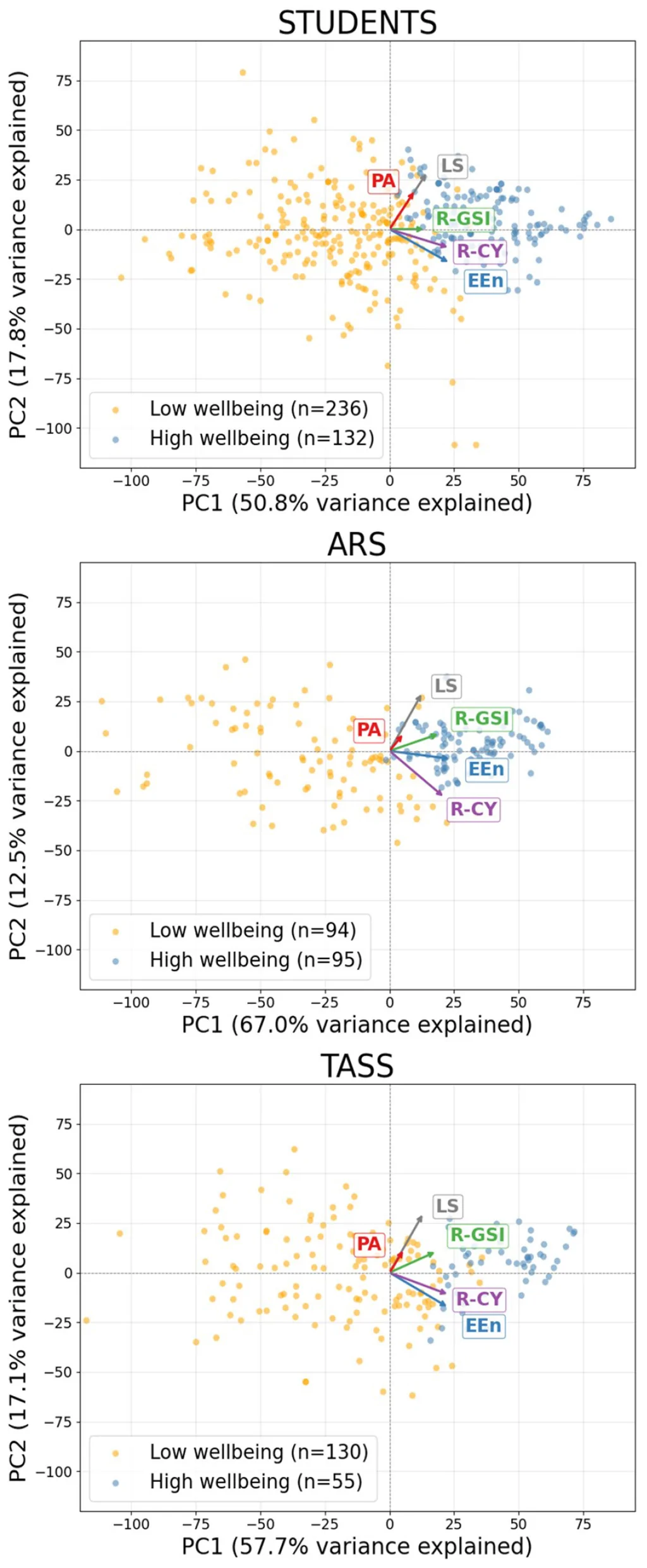
Principal component biplot with LPA clusters for the three university groups. Points show participants by the first two principal components. Color indicates wellbeing: orange (lower) and blue (higher). Arrows show the loadings of each indicator in the factorial space. R-GSI, Global Severity Index (reverse-coded); EEn, Emotional Energy; R-CY, Reversed Cynicism; PA, Personal Accomplishment; LS, Life Satisfaction.

A Latent Profile Analysis (LPA) was conducted independently for each university collective: Students, ARS, and TASS. Since the input variables were continuous and the final assignment was deterministic (hard classification), the probabilistic evaluation was complemented with geometric separation metrics, allowing the assessment of both internal cohesion and distinction among the resulting profiles. Figure 3 shows the principal component biplot with LPA clusters for the three university groups.

The selection of the number of classes was based on a multi-criteria approach combining probabilistic fit (BIC/AIC), classification accuracy (entropy, AvePP), geometric separation (Silhouette), and solution stability (ARI; see Table 8).

**Table 8:** LPA model selection indices by group and number of classes (K).

Group	K	Log-L	AIC	BIC	Entropy	AvePPmin	Silhouette	Class sizes (n)	ARI
Students	2	–8,136.9	16,315.8	16,397.9	0.79	0.92	0.26	[132, 236]	1.00 ± 0.00
	3	–8,080.3	16,224.5	16,349.6	0.78	0.83	0.22	[206, 45, 117]	0.97 ± 0.11
	4	–7,634.1	15,354.1	15,522.2	0.84	0.84	0.10	[56, 83, 178, 51]	0.53 ± 0.32
	5	–7,598.0	15,304.1	15,515.1	0.88	0.84	0.12	[56, 7, 176, 45, 84]	0.53 ± 0.12
	6	–7,561.4	15,252.8	15,506.9	0.85	0.82	0.09	[78, 40, 43, 56, 144, 7]	0.83 ± 0.16
ARS	2	–4,007.0	8,056.0	8124.0	0.87	0.96	0.34	[94, 95]	1.00 ± 0.00
	3	–3,954.3	7,972.6	8076.4	0.84	0.92	0.26	[75, 37, 77]	1.00 ± 0.00
	4	–3,933.6	7,953.2	8092.6	0.83	0.88	0.16	[33, 70, 62, 24]	0.70 ± 0.22
	5	–3,912.3	7,932.5	8107.6	0.81	0.79	0.16	[22, 34, 58, 17, 58]	0.70 ± 0.22
	6	–3,904.8	7,939.6	8150.3	0.87	0.86	0.17	[32, 66, 11, 52, 26, 2]	0.56 ± 0.11
TASS	2	–4,026.9	8,095.8	8,163.4	0.88	0.94	0.25	[130, 55]	1.00 ± 0.00
	3	–3,977.1	8,018.3	8,121.3	0.83	0.90	0.20	[73, 31, 81]	0.91 ± 0.11
	4	–3,953.9	7,993.9	8,132.4	0.88	0.92	0.22	[31, 66, 77, 11]	0.66 ± 0.26
	5	–3,919.7	7,947.4	8,121.3	0.87	0.90	0.15	[60, 11, 23, 63, 28]	0.28 ± 0.06
	6	–3,676.5	7,483.0	7,692.3	0.86	0.76	0.07	[33, 33, 45, 25, 24, 25]	0.74 ± 0.20

Log-L, log-likelihood; AIC, Akaike Information Criterion; BIC, Bayesian Information Criterion; AvePPmin, minimum average posterior probability; ARI, Adjusted Rand Index (mean ± SD across 5-fold cross-validation).

Regarding probabilistic fit, AIC and BIC decreased monotonically in Students and TASS, common pattern in LPA with non-normally distributed continuous indicators (Nylund et al., 2007), so guidelines recommend prioritizing entropy, AvePP, and interpretability over isolated BIC/AIC values (Masyn, 2013). In ARS, BIC reached a minimum at K = 3 (ΔBIC = 48 relative to K = 2), but increased again for K ≥ 4, indicating that the marginal improvement did not hold with greater complexity. In response to the reviewer's concern that rejection of K = 3 was not sufficiently justified, we present below the complete K = 3 solutions for all three groups, together with direct evidence on profile interpretability, partition structure, and empirical stability (see Table 9).

Table 9Complete K = 2 and K = 3 solutions: class-specific indicator means, classification quality, partition correspondence, and stability across 20 random initialisations.

**Table:** 

Panel A. Classification quality and stability						
	Students		ARS		TASS	
	K = 2	K = 3	K = 2	K = 3	K = 2	K = 3
Relative entropy	0.793	0.777	0.870	0.844	0.884	0.832
AvePP (minimum class)	0.916	0.828	0.957	0.920	0.939	0.899
ARI: *M* (*SD*)	1.000 (0.000)	0.974 (0.111)	1.000 (0.000)	1.000 (0.000)	1.000 (0.000)	0.910 (0.111)
ARI minimum	1.000	0.488	1.000	1.000	1.000	0.774
ARI (K = 2 vs K = 3)	0.722		0.465		0.335	
**Panel B. K = 2 class profiles**						
	**Students**		**ARS**		**TASS**	
	**Low**	**High**	**Low**	**High**	**Low**	**High**
*n*	236	132	94	95	130	55
(%)	(64.1%)	(35.9%)	(49.7%)	(50.3%)	(70.3%)	(29.7%)
R-GSI	36.66	55.77	46.58	78.52	50.99	80.68
EEn	30.01	59.80	43.16	76.81	51.90	84.73
R-CY	58.19	90.53	59.18	90.88	62.08	96.74
PA	62.38	83.19	78.66	90.06	78.80	92.42
LS	48.58	74.82	56.20	81.93	54.37	77.04
AvePP	0.954	0.916	0.972	0.957	0.977	0.939

**Table:** 

Panel C. K = 3 class profiles									
	Students			ARS			TASS		
	Low	Mid	High	Low	Mid	High	Low	Mid	High
*n*	206	45	117	37	77	75	81	73	31
(%)	(56.0%)	(12.2%)	(31.8%)	(19.6%)	(40. 7%)	(39.7%)	(43.8%)	(39.5%)	(16.8%)
R-GSI	32.72	**62.92** ^†^	55.07	34.84	58.71	80.38	46.26	61.60	91.06
EEn	24.76	**66.00** ^†^	59.03	19.10	**61.86** ^‡^	78.44	41.36	71.87	90.65
R-CY	57.42	68.33	92.13	38.40	**74.30** ^‡^	94.06	50.10	**86.02** ^‡^	98.52
PA	64.85	53.46	84.95	76.95	80.30	92.26	74.38	87.71	93.55
LS	48.17	60.47	74.33	52.97	63.74	82.64	53.22	60.44	83.29
AvePP	0.928	0.828	0.893	0.948	0.937	0.920	0.937	0.899	0.953
*Mid-class origin (subjects drawn from K = 2):*									
From K = 2 Low	—	33	—	—	57	—	—	49	—
From K = 2 High	—	12	—	—	20	—	—	24	—

All scores on 0–100 scale. R-GSI, Global Severity Index (reverse-coded; higher = better); EEn, Emotional Energy (higher = less exhausted); R-CY, Reversed Cynicism (higher = less cynicism); PA, Personal Accomplishment; LS = Life Satisfaction. AvePP, Average Maximum Posterior Probability. ARI, Adjusted Rand Index; ARI (20 seeds) = replication across random initialisations; ARI (K = 2 vs K = 3), correspondence between partitions for the same sample. ^†^ Mid-class scores exceed the High-wellbeing class, violating the expected monotonic gradient. ^‡^ Mid-class scores show non-linear elevation relative to the linear interpolation between Low and High classes.

In terms of classification accuracy, K = 2 maximized entropy across all three groups (Students: 0.793; ARS: 0.870; TASS: 0.884). AvePP exceeded the standard threshold of 0.70 in all classes for K = 2 (Students: 0.92; ARS: 0.96; TASS: 0.94). For K = 3, entropy and AvePP remained acceptable in all groups (Table 9); accordingly, we do not base rejection of K = 3 on these indices alone.

Geometric separation was consistent: the Silhouette index was highest at K = 2 across all groups (Students: 0.26; ARS: 0.34; TASS: 0.25) and decreased when moving to K = 3 (0.22; 0.26; 0.20, respectively). For K ≥ 4, the index collapsed in all groups (< 0.17), indicating that additional classes capture noise rather than structurally distinct profiles.

The critical evidence for preferring K = 2 over K = 3 rests on three convergent findings detailed below; the full K = 3 solutions are presented in Table 9.

First, in no group does the intermediate K = 3 class produce a theoretically coherent “moderate flourishing” profile in the sense of Keyes (2002, 2007). In Students, the intermediate class (*n* = 45, 12.2%) scored higher on R-GSI (62.92) and emotional exhaustion (EEn = 66.00) than the High-wellbeing class (R-GSI = 55.07, EEn = 59.03), violating the monotonic gradient expected from a three-point wellbeing continuum and indicating a burnout-specific pattern rather than a transitional wellbeing state. In ARS, the intermediate class (*n* = 77, 40.7%) showed disproportionate elevation of EEn and R-CY relative to the linear interpolation between the Low and High classes (residuals of 13.09 and 8.07 points, respectively), consistent with an exhaustion–cynicism configuration rather than moderate flourishing. In TASS, the intermediate class (*n* = 73, 39.5%) similarly showed non-linear elevation of P (residual = 11.70 points) and LS (7.82 points), producing a profile inconsistent with Keyes' intermediate category. It should be noted that Keyes' flourishing–moderate–languishing typology is derived from categorical diagnostic criteria applied to hedonic and eudaimonic indicators, not from continuous-indicator LPA; consequently, a direct mapping between the two frameworks is methodologically untenable (Lubke and Muthén, 2005; Masyn, 2013).

Second, in all three groups the K = 3 partition constitutes a restructuring of the K = 2 boundary rather than the identification of a new nested subgroup. In Students, the intermediate K = 3 class drew 33 subjects from the K = 2 Low-wellbeing class and 12 from the K = 2 High-wellbeing class (ARI[K = 2, K = 3] = 0.722). In ARS, the intermediate class (*n* = 77) comprised 57 subjects from the K = 2 Low class and 20 from the K = 2 High class (ARI[K = 2, K = 3] = 0.465), meaning that 61% of participants originally in the Low class would be reclassified as “intermediate” a wholesale redefinition of the low-wellbeing group rather than identification of a distinct subgroup within it. In TASS, ARI[K = 2, K = 3] = 0.335 near chance, indicating that K = 2 and K = 3 produce nearly independent partitions of the same data.

Third, solution stability across 20 random initialisations (Table 9) showed that K = 2 replicated perfectly in all three groups (ARI = 1.000, *SD* = 0.000). In Students, K = 3 showed substantial instability (ARI min = 0.488), with one initialization producing near-random correspondence to the reference solution. In TASS, K = 3 showed moderate instability (ARI min = 0.774, *SD* = 0.111). In ARS, K = 3 was perfectly stable (ARI = 1.000, *SD* = 0.000); here, rejection rests entirely on the profile interpretability and partition restructuring arguments above.

Overall, K = 2 simultaneously maximized entropy, AvePP, and geometric separation, showed perfect stability across initialisations, and yielded interpretable profiles (high vs. low wellbeing) in all three groups. The K = 3 intermediate class did not produce a theoretically coherent moderate-wellbeing profile in any group, and the K = 3 partition restructured the K = 2 boundary rather than revealing new latent subgroups.

These considerations, taken together with the theoretical and measurement-level incompatibility between Keyes' categorical typology and continuous-indicator LPA, justify retaining K = 2 as the optimal solution across all groups.

Each point represents a participant projected onto the first two principal components, with color indicating cluster membership (orange = lower wellbeing; blue = higher wellbeing). Arrows indicate the loadings of each indicator in the factorial space.

The first two components (PC1 and PC2) explained the following variance: Students (*n* = 368): PC1 = 50.8%, PC2 = 17.8%; ARS (*n* = 189): PC1 = 67.0%, PC2 = 12.5%; TASS (*n* = 185): PC1 = 57.7%, PC2 = 17.1%. Among Students, a moderate separation between profiles was observed, with 64.1% in the lower wellbeing cluster. In the ARS group, the proportion between clusters was balanced, with moderate separation and some central overlap; 49.7% belonged to the lower wellbeing cluster. In the TASS group, the distribution was highly unbalanced, with 70.3% in the lower wellbeing cluster.

To characterize the internal structure of the identified profiles, the five wellbeing indicators were compared across clusters using the Mann–Whitney test and Cohen's d effect sizes. Since these indicators were also used to derive the profiles, the observed differences are expected by construction and should be interpreted as descriptive characterizations of the profiles rather than as independent validation of the cluster solution. The results, presented in Table 10, showed that in all three population groups (Students, ARS, and TASS), all variables exhibited statistically significant differences [Cohen's d ranging from 0.975 to 2.019, (*p* < 0.001) across all groups], reflecting the internal differentiation of the profiles across all assessed dimensions.

**Table 10:** Comparison of latent classes by variable and group.

Variable	Group	Cluster 0 (*M*)	Cluster 1 (*M*)	*U* (*p*)	*d* (95% CI)
R-GSI	Students	36.66	55.77	< 0.001	1.139 (0.91, 1.38)
	ARS	46.58	78.52	< 0.001	2.019 (1.74, 2.35)
	TASS	50.99	80.68	< 0.001	1.751 (1.40, 2.16)
EEn	Students	30.01	59.80	< 0.001	1.331 (1.09, 1.59)
	ARS	43.16	76.81	< 0.001	1.650 (1.35, 2.03)
	TASS	51.90	84.73	< 0.001	1.691 (1.38, 2.03)
R-CY	Students	58.19	90.53	< 0.001	1.609 (1.44, 1.82)
	ARS	59.18	90.88	< 0.001	1.606 (1.34, 1.94)
	TASS	62.08	96.74	< 0.001	1.931 (1.71, 2.19)
PA	Students	62.38	83.19	< 0.001	1.395 (1.19, 1.62)
	ARS	78.66	90.06	< 0.001	0.975 (0.70, 1.27)
	TASS	78.80	92.42	< 0.001	1.001 (0.77, 1.27)
LS	Students	48.58	74.82	< 0.001	1.509 (1.30, 1.75)
	ARS	56.20	81.93	< 0.001	1.762 (1.47, 2.10)
	TASS	54.37	77.04	< 0.001	1.223 (0.91, 1.55)

Values are reported as mean per cluster (Cluster 0 and Cluster 1), the *p*-value of the Mann–Whitney test (*U* *p*), the effect size using Cohen's *d* and the 95% confidence interval (CI). The variable R-GSI corresponds to Global Severity Index (reverse-coded), EEn to Emotional Energy, R-CY to Reversed Cynicism, PA to Personal Accomplishment, and LS to Life Satisfaction.

In the student group, differences between clusters were substantial across all dimensions: Cluster 1 (higher wellbeing) scored higher than Cluster 0 on R-GSI (55.77 vs. 36.66; d = 1.139), EEn (59.80 vs. 30.01; d = 1.331), R-CY (90.53 vs. 58.19; d = 1.609), PA (83.19 vs. 62.38; d = 1.395), and LS (74.82 vs. 48.58; d = 1.509), with R-CY showing the largest difference.

In the ARS group, the most pronounced discrepancies were observed: R-GSI had the largest effect size (78.52 vs. 46.58; d = 2.019), followed by LS (d = 1.762), EEn (d = 1.650), R-CY (d = 1.606), and PA (d = 0.975). The TASS group exhibited a pattern similar to ARS, with R-GSI (d = 1.751) and R-CY (96.74 vs. 62.08; d = 1.931) showing the strongest effects, while LS (d = 1.223) and PA (d = 1.001) also displayed significant differences, the latter being the smallest.

Before interpreting the contribution of individual variables to cluster formation, the internal coherence of the Random Forest model was evaluated using cross-validation, extracting global performance metrics (AUC, accuracy, recall/sensitivity, and precision), presented in Supplementary Table S1.

It must be noted that this model was trained and evaluated on the same five variables used to derive the profiles; consequently, the near-perfect AUC values obtained (≥ 0.986) are an expected consequence of this design and do not constitute external validation of the cluster solution. These results are intended solely as a complementary descriptive tool to illustrate the relative contribution of each indicator to the internal differentiation of the profiles, not to assess their robustness or generalizability.

The consistently high performance across all three groups (AUC ≥ 0.986) reflects the circular nature of the procedure, given that the classifier was trained on the same variables defining the clusters. These metrics therefore describe the internal coherence of the profiles and should not be interpreted as evidence of their external validity. They are reported here to support the interpretability of the variable importance scores used to construct the radar charts.

**Figure 4:**
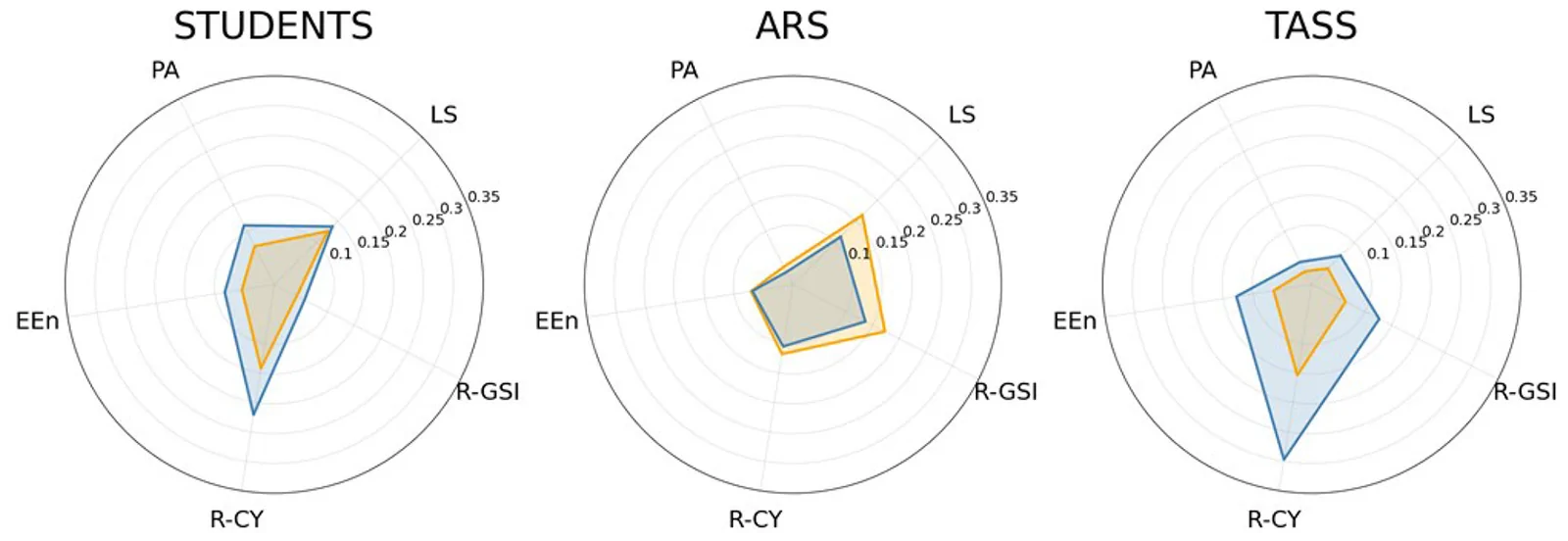
Radar charts displaying for each wellbeing indicator across the three university groups. Each axis represents one indicator; the shaded area reflects its relative contribution to cluster discrimination. Orange = lower wellbeing cluster; blue = higher wellbeing cluster. R-GSI, Global Severity Index (reverse-coded); EEn, emotional energy; R-CY, Reversed Cynicism; PA, Personal Accomplishment; LS, life satisfaction.

The radar charts (Figure 4) display the relative importance of each wellbeing variable in discriminating between clusters within each group, derived from mean absolute SHAP values computed per cluster using the trained Random Forest model. Each polygon represents one cluster (orange = lower wellbeing; blue = higher wellbeing), and its extension along each axis reflects the mean absolute SHAP contribution of that indicator to the classification of participants within that cluster. These values are intended as an exploratory internal visualization and do not constitute external validation of the cluster solution.

In the Student group, the most notable separation between clusters was observed along the R-CY (Reversed Cynicism) and PA (Personal Accomplishment) axes: the lower wellbeing cluster showed greater extension toward cynicism, whereas the higher wellbeing cluster extended more prominently toward personal accomplishment. LS showed the least separation between clusters, with both polygons reaching similar extensions along this axis. EEn and R-GSI showed moderate contributions, with slightly greater extension in the lower wellbeing cluster.

In the ARS group, the most notable separation between clusters was observed along the R-GSIs and LS (life satisfaction) axes: the lower wellbeing cluster showed greater extension toward psychological distress, whereas the higher wellbeing cluster extended somewhat more toward life satisfaction. In contrast, R-CY and EEn showed the least differentiation between clusters, with both polygons reaching similar extensions along these axes. PA showed a moderate contribution, with slightly greater extension in the lower wellbeing cluster.

In the TASS group, the separation between clusters was the most pronounced of the three groups. The higher wellbeing cluster showed clearly greater extension, particularly along the R-CY, EEn, and R-GSI axes, while the lower wellbeing cluster was notably more compact. This pattern indicates that in TASS, cluster membership is primarily driven by Reversed Cynicism (R-CY) and Emotional Energy (EEn), with R-GSI also playing a relevant discriminative role.

### 3.2 Predictors of cluster membership

In the logistic regression analysis conducted for the three groups, the dependent variable was membership in the Wellbeing cluster (dichotomous variable: Cluster 0 = low wellbeing vs. Cluster 1 = high wellbeing). Independent variables were selected through a two-step process: in the exploratory phase, bivariate associations between each sociodemographic/health habit variable Table 1 and the clusters were assessed using χ^2^ tests and Cramér's V; in the modeling phase, variables with significant associations (*p* < 0.05) and/or theoretical relevance were included in the multivariate logistic regression model, reporting β coefficients, odds ratios (OR) with 95% confidence intervals, and p-values. Subsequently, a multivariate logistic regression analysis was conducted separately for each of the three groups, simultaneously including Self-Perceived Health, Economic Situation, Use of Tranquilizers/Hypnotics, Sleep Quality, Drug Consumption, Sports Practice, Hours of Physical Activity, and Gender as predictors.

Prior to interpreting the regression results, the linearity assumption for the two ordinal predictors was examined via likelihood ratio tests comparing the linear specification against an unrestricted dummy-coded version in each group. The assumption was supported for economic situation across all three groups (Students: LRT χ^2^(2) = 1.94, *p* = 0.378; ARS: LRT χ^2^(2) = 2.42, *p* = 0.299; TASS: LRT χ^2^(2) = 5.43, *p* = 0.066) and for self-perceived health in Students (LRT χ^2^(3) = 0.54, *p* = 0.911) and ARS (LRT χ^2^(2) = −1.07, *p* = 1.000). In the TASS group, the LRT for self-perceived health yielded a nominally significant result (LRT χ^2^(3) = 11.25, *p* = 0.010); however, this should be interpreted with caution, as the dummy-coded model failed to converge and two response categories contained extremely small cell sizes (*n* = 2 and *n* = 9), which precluded stable parameter estimation and likely inflated the test statistic. Inspection of raw proportions across categories confirmed a monotonic trend fully consistent with linearity (proportions of high wellbeing: 0%, 7%, 28%, and 57% across categories 2–5). The linear specification was therefore retained across all groups; however, given the convergence failure and the extremely small cell sizes in two categories, the linearity assumption for self-perceived health in the TASS group should be regarded as provisional and interpreted with caution.

Table 11 presents the complete results of the binary logistic regression model for each group, including logistic coefficients (β), standard errors (SE), odds ratios (OR) with 95% confidence intervals, *p*-values, and model fit indices (McFadden and Nagelkerke pseudo-*R*^2^, AIC, BIC). Model calibration was assessed using the Hosmer–Lemeshow test and discriminative ability via the area under the ROC curve (AUC). Based on the variables listed in Table 1, the logistic regression model retained five predictors across groups: Self-Perceived Health, Economic Situation, Use of Tranquilizers/Hypnotics, Sleep Quality, and Gender.

**Table 11:** Multivariate logistic regression predicting wellbeing cluster membership by group.

Students (*n* = 365)					
Variable	β	SE	OR	95% CI	*p*
Constant	5.80	1.35	330.07	[23.47, 4642.18]	< 0.001^***^
Self-perceived health	−0.99	0.22	0.37	[0.24, 0.57]	< 0.001^***^
Economic situation	−0.48	0.19	0.62	[0.43, 0.89]	0.010^*^
Tranquilizers/hypnotics	0.99	0.37	2.68	[1.30, 5.51]	0.007^**^
Sleep quality	−0.73	0.26	0.48	[0.29, 0.81]	0.005^**^
Gender	−1.00	0.31	0.37	[0.20, 0.67]	0.001^**^
*Model fit: McFadden *R*^2^ = 0.202, Nagelkerke *R*^2^ = 0.319, AIC = 400.05, BIC = 439.04*					
*AUC-ROC = 0.788, H-L χ^2^(8) = 3.74 (*p* = 0.879), Log-lik. = −190.02, LRT *p* < 0.001*					
**ARS (*n* = 182)**					
**Variable**	**β**	**SE**	**OR**	**95% CI**	***p***
Constant	10.15	2.17	25600.58	[367.28, 1784462]	< 0.001^***^
Self-perceived health	−0.35	0.30	0.70	[0.39, 1.26]	0.234
Economic situation	−1.18	0.29	0.31	[0.17, 0.54]	< 0.001^***^
Tranquilizers/hypnotics	−1.42	0.54	0.24	[0.08, 0.71]	0.009^**^
Sleep quality	−0.94	0.38	0.39	[0.18, 0.82]	0.013^*^
Gender	−0.88	0.38	0.42	[0.20, 0.88]	0.022^*^
*Model fit: McFadden *R*^2^ = 0.232, Nagelkerke *R*^2^ = 0.367, AIC = 213.80, BIC = 245.84*					
*AUC-ROC = 0.808, H-L χ^2^(8) = 4.58 (*p* = 0.801), Log-lik. = −96.90, LRT *p* < 0.001*					
**TASS^⊺^(*n* = 180)**					
**Variable**	**β**	**SE**	**OR**	**95% CI**	***p***
Constant	6.94	2.02	1034.90	[19.66, 54488.36]	< 0.001^***^
Self-perceived health	−0.60	0.26	0.55	[0.33, 0.91]	0.020^*^
Economic situation	−1.34	0.34	0.26	[0.14, 0.50]	< 0.001^***^
Tranquilizers/hypnotics	−0.58	0.56	0.56	[0.19, 1.67]	0.297
Sleep quality	−0.63	0.44	0.53	[0.23, 1.25]	0.149
Gender	0.74	0.44	2.09	[0.89, 4.92]	0.090
*Model fit: McFadden *R*^2^ = 0.323, Nagelkerke *R*^2^ = 0.461, AIC = 167.82, BIC = 199.75*					
*AUC-ROC = 0.815, H-L χ^2^(8) = 5.76 (*p* = 0.673), Log-lik. = −73.91, LRT *p* < 0.001*					

β, logistic coefficient; SE, standard error; OR, odds ratio; 95% CI, confidence interval for OR. Full model includes 9 predictors; only selected variables shown. Reference categories: Gender: 0 = male; Tranquilizers: 0 = non-user; Sleep quality: 1 = good quality. ^⊺^TASS estimates obtained via Firth penalized-likelihood logistic regression due to complete separation in the standard model. ^*^ *p* < 0.05 ^**^ *p* < 0.01 ^***^p < 0.001. Odds ratios below 1 indicate a reduced likelihood of membership in the low wellbeing cluster (Cluster 0), and odds ratios above 1 indicate an increased likelihood, consistent with the outcome coding described in Section 2.6.

As a further sensitivity check, we re-estimated the TASS model with the two sparse self-perceived health categories responsible for the convergence failure (*n* = 2 and *n* = 9) merged into a single category. The effect of self-perceived health remained significant and of comparable magnitude (OR = 0.48, 95% CI [0.27, 0.84], *p* = 0.011), indicating that the result reported in Table 11 does not hinge on these unstable cells. A more aggressive three-category collapse that additionally merged the well-populated “good” and “very good” categories (*n* = 106 and *n* = 35) attenuated the effect to a marginal level (OR = 0.44, 95% CI [0.18, 1.07], *p* = 0.069); given the size and distinct wellbeing profiles of these two categories, this attenuation reflects loss of genuine variation rather than dependence on sparse cells.

These sample sizes correspond to the latent profile analysis and differ slightly from those used in the logistic regression analysis, where the exclusion of cases with missing values in sociodemographic variables reduced the sample to Students (*n* = 365), ARS (*n* = 182), and TASS (*n* = 180).

Model fit was adequate for each group (Students: McFadden R^2^ = 0.202, Nagelkerke R^2^ = 0.319, *p* < 0.001; ARS: McFadden R^2^ = 0.232, Nagelkerke R^2^ = 0.367, *p* < 0.001; TASS: McFadden R^2^ = 0.323, Nagelkerke R^2^ = 0.461, *p* < 0.001), indicating pseudo-R^2^ values reflecting moderate-to-substantial explanatory strength for profile membership, ranging between 20% and 32% according to the more conservative McFadden index, and between 32% and 46% according to Nagelkerke R^2^. Discriminatory ability was high across all groups (AUC-ROC: Students = 0.788, ARS = 0.808, TASS = 0.815), and Hosmer–Lemeshow tests indicated adequate calibration in all cases (*p* > 0.05).

In students (*n* = 365), five variables significantly predicted membership in the low wellbeing cluster (Cluster 0). Self-Perceived Health was the strongest predictor (OR = 0.37, *p* < 0.001), reducing the likelihood of low wellbeing by 63% for each unit increase. Gender showed a protective effect for women (OR = 0.37, *p* = 0.001), which was not attributable to the sample imbalance (ratio 2.3:1) but confirmed as a true effect by standardized residual analysis (deficit of 10.5 women in Cluster 0; χ^2^ = 5.63, *p* = 0.018, *V* = 0.124). Use of Tranquilizers/Hypnotics increased the probability of low wellbeing by 168% (OR = 2.68, *p* = 0.007). Better Economic Situation reduced the risk by 38% per level of improvement (1–4 scale; OR = 0.62, *p* = 0.010). Adequate Sleep Quality decreased the probability of low wellbeing by 52% (OR = 0.48, *p* = 0.005). Drug use, sports practice, and hours of physical activity did not reach statistical significance.

In the ARS group (*n* = 182), multivariate logistic regression identified four significant predictors of psychological wellbeing. Tranquilizers/Hypnotics use was the most relevant factor (β = −1.42, *p* = 0.009; OR = 0.24; 95% CI: [0.08, 0.71]), such that not using them reduced the likelihood of belonging to the low wellbeing cluster by 76%. Economic Situation showed a robust effect (β = −1.18, *p* < 0.001; OR = 0.31; 95% CI: [0.17, 0.54]), while Sleep Quality was protective (β = −0.94, *p* = 0.013; OR = 0.39; 95% CI: [0.18, 0.82]), with this effect particularly pronounced in ARS. Gender was also significant in the adjusted model (β = −0.88, *p* = 0.022; OR = 0.42; 95% CI: [0.20, 0.88]), although no significant association was observed in the bivariate analysis. This pattern may reflect a suppression effect arising from the inclusion of other covariates in the model (e.g., economic situation or use of Tranquilizers/Hypnotics). Accordingly, this result should be interpreted with caution, and its substantive meaning remains tentative. Self-Perceived Health, drug use, sports practice, and hours of physical activity did not reach statistical significance.

In the TASS group (*n* = 180), standard logistic regression failed to converge due to complete separation caused by the drug use variable: all seven participants who reported drug use belonged to Cluster 0 (lower wellbeing), which prevents the likelihood function from having a finite maximum and causes the associated coefficient to tend toward infinity. To address this, Firth penalized logistic regression was applied, which introduces a Jeffreys prior penalty on the likelihood function. This method produces finite, reduced-bias coefficient estimates even in the presence of perfect or quasi-perfect separation, without requiring variable exclusion or ad hoc regularization procedures. All remaining model coefficients also benefit from this correction; therefore, all results presented for the TASS group correspond entirely to this penalized estimation.

The analysis in TASS group, identified two significant predictors of membership in the low psychological wellbeing cluster. Economic hardship emerged as the strongest factor (β = −1.344, *p* < 0.001; OR = 0.26; 95% CI: [0.14, 0.50]), such that each improvement in economic status reduced the probability of low wellbeing by 74%. Additionally, better Self-Perceived Health showed a significant protective effect (β = −0.603, *p* = 0.020; OR = 0.55; 95% CI: [0.33, 0.91]), reducing the risk by 45% for each unit increase in self-perceived health. In contrast, Tranquilizers/Hypnotics use (β = −0.58, *p* = 0.297; OR = 0.56; 95% CI: [0.19, 1.67]) and Sleep Quality (β = −0.63, *p* = 0.149; OR = 0.53; 95% CI: [0.23, 1.25]) showed directional effects consistent with the other groups but did not reach statistical significance, possibly due to limited statistical power in this sub-sample. Gender (β = 0.74, *p* = 0.090; OR = 2.09; 95% CI: [0.89, 4.92]), by contrast, reversed direction relative to Students and ARS, where it was protective for women. This reversal most plausibly reflects the smaller and more imbalanced male reference category in TASS (*n* = 48, 25.9% of the group, the most skewed sex ratio of the three groups) together with the wide confidence interval spanning near-null values, rather than a confirmed genuine group difference; it should therefore be interpreted with caution pending replication in larger samples.

In this group, drug use, which had previously been excluded due to complete separation, could be included using Firth's regression. It showed a large directional effect (β = 1.940; OR = 6.96), indicating an increased risk of belonging to the low wellbeing cluster, although it did not reach statistical significance (*p* = 0.264) due to the small number of cases. Notably, all participants who reported drug use were in the low wellbeing cluster, which can be considered a qualitative indicator of extreme risk, relevant for future studies with larger sample sizes. Sports practice and hours of physical activity did not reach statistical significance in this group.

Overall, these findings suggest the relevance of economic status and Self-Perceived Health as predictors of psychological wellbeing in TASS and highlight the usefulness of the Firth penalized method for examining variables with extreme distributions or complete separation, such as Drug Use.

## 4 Discussion

### 4.1 Key findings

The results suggest the presence of two psychological wellbeing profiles (low vs. high) across the three groups analyzed, with a high proportion of individuals in the lower wellbeing profile, particularly among TASS and students. Because the present study operationalized global psychological wellbeing as a unidimensional composite factor integrating reversed psychological distress, emotional energy, reversed cynicism, personal accomplishment, and life satisfaction, these profiles should be interpreted as reflecting relative positions along an overall wellbeing continuum. Accordingly, the findings should not be read as a direct test of the dual-continua model of mental health, which conceptualizes wellbeing and distress as related but distinct dimensions. Rather, they are broadly consistent with dimensional approaches suggesting that psychological symptoms and positive functioning may combine in different ways within university populations (Keyes, 2002; Schaufeli et al., 2002; Li et al., 2022).

### 4.2 Comparison with previous studies

From the “stratified wellbeing” perspective, the findings reinforce the idea of the university as an organizational field in which the structural position of each group translates into distinct patterns of vulnerability and protection (Bourdieu, 1988; Marginson, 2008). The higher concentration of low wellbeing among TASS and Students suggests that positions with less control over work or study conditions, lower institutional recognition, and greater economic uncertainty are associated with higher psychological risk, as proposed by job demands–resources models (Bakker and Demerouti, 2007; Standing, 2011). In contrast, the more polarized profile observed in ARS is compatible with the coexistence of relatively stable career trajectories alongside others characterized by instability and productivity pressure (Mudrak et al., 2018; Cadena-Povea et al., 2025).

The identification of Economic Status as a consistent structural predictor across all groups, together with the significant contribution of Self-Perceived Health among Students (*p* < 0.001) and TASS (*p* = 0.020), though not among ARS (OR = 0.70, *p* = 0.234), aligns with the literature highlighting the role of social and material determinants in mental health, even in highly qualified contexts such as universities (Kelloway et al., 2023; World Health Organization, 2022c). At the same time, the differential weight of Reversed Cynicism in Students and TASS, and of global psychological distress (R–GSI) in ARS, is consistent with recent evidence positioning cynicism as a central and potentially precursor component of burnout (Schaufeli, 2003), while emphasizing the role of connection and belonging in the experience of wellbeing in higher education (Corpuz, 2023).

Overall, the present study contributes to shifting the focus from isolated indicators (e.g., burnout alone or psychological distress symptoms alone) toward a broader construct of global psychological wellbeing that integrates symptoms, emotional energy, reversed cynicism, personal accomplishment, and life satisfaction, in line with more recent dimensional approaches (De Beer et al., 2024; Vázquez et al., 2013). Furthermore, the combination of Latent Profile Analysis (LPA) and machine learning–based classification models advances toward an applied psychometric framework that not only describes average trends, but also identifies practically informative risk profiles.

### 4.3 Practical and institutional implications

At the applied level, the results highlight the need for stratified intervention strategies rather than homogeneous approaches for the entire university community. In TASS, the high prevalence of low wellbeing, together with the central role of economic status and self-perceived health, is consistent with primarily structural vulnerability, in line with studies documenting precariousness, organizational cynicism, and lower recognition in this group (Akbas et al., 2018; Coleman and Ali, 2025; Bruno et al., 2024). This pattern suggests that the most promising interventions may combine improvements in contractual stability, working conditions, and participation in decision-making with targeted psychosocial support resources.

Among Academic and Research Staff (ARS), the lower wellbeing profile was characterized by higher global psychological distress and was associated with anxiolytic, antidepressant, or hypnotic/sedative medication use and poorer sleep quality, consistent with the cumulative impact of sustained demands related to productivity, accreditation, and bureaucratic workload (Mudrak et al., 2018; Jayman et al., 2022). The relevance of sleep quality is also supported by evidence modeling sleep quality as a quantitative predictor of mental wellbeing variance, although this evidence comes from older adults during the COVID-19 lockdown rather than from a university community sample (Trabelsi et al., 2021). These findings align with studies highlighting faculty overload and the need for workload management and occupational health policies specific to this group (Kinman and Wray, 2013; Innstrand and Christensen, 2018). A dual-axis intervention approach may therefore be warranted: reducing non-essential structural demands and implementing programs that promote healthy habits, prioritizing rest and non-pharmacological stress management alternatives.

Among students, the co-occurrence of poorer economic status, lower self-perceived health, psychotropic medication use, and gender differences observed in the present study is consistent with peer-reviewed evidence documenting substantial levels of anxiety, depression, psychological distress, and academic burnout in university populations, including first-year students (March-Amengual et al., 2022; Li et al., 2022). This pattern suggests the potential value of multicomponent university transition support combining financial and academic assistance with evidence-based psychological interventions, emotion-regulation support, and facilitated access to mental health services. This interpretation is consistent with institutional recommendations and peer-reviewed evidence on interventions and factors supporting mental health and academic adjustment in higher education (Worsley et al., 2022; Kritikou and Giovazolias, 2022; Departament de Recerca i Universitats, 2023).

Although sports practice and hours of physical activity were not significant predictors in the present models, these null findings should be interpreted cautiously. Prior evidence in university students suggests that physical activity is associated with lower negative emotional states, including depression, anxiety, and stress, and may operate within broader pathways involving problematic internet use (Jelleli et al., 2024). This pattern is also consistent with more recent machine-learning evidence: in a large community sample using a comparable Random Forest-based framework combining psychological distress, digital behavioral risk, and physical activity as joint predictors of subjective wellbeing, physical activity showed a consistent but comparatively modest protective association once psychological distress was included, with psychological distress emerging as the dominant predictor (Saidane et al., 2026). In the present study, the lack of significant associations may reflect the broad operationalization of physical activity, which was captured through sports practice and weekly activity hours, as well as possible residual confounding or attenuation after adjustment for other relevant predictors, such as economic situation, self-perceived health, medication use, sleep quality, and gender. Accordingly, these results should not be taken as evidence that physical activity is unrelated to wellbeing, but rather as indicating that its role may require more specific measurement and longitudinal examination.

Across all groups, the consistent association of economic status with wellbeing profiles, reinforced by the additional contribution of perceived health among Students and TASS, suggests that university wellbeing policies may benefit from being articulated alongside social protection and income support strategies, beyond interventions focused exclusively on individual resilience (Kelloway et al., 2023; World Health Organization, 2022b). The results of this study are consistent with the need for multilevel approaches combining individual interventions (e.g., socio-emotional skills training, wellbeing programs) with organizational changes and university policy decisions (scholarships, contracts, and workload), in line with healthy university frameworks and comprehensive campus health promotion programs (Innstrand and Christensen, 2018; Corpuz, 2023).

Finally, the exploratory methodological approach employed, based on latent profiles and multivariable analysis, may offer a useful starting point for other institutions seeking to examine wellbeing heterogeneity within their communities. However, replication in independent samples and external validation would be necessary before considering its use for applied screening, resource allocation, or intervention-planning purposes (Jayman et al., 2022).

### 4.4 Strengths and limitations

Mental health in the university context cannot be addressed solely in a fragmented or individualized manner. The results of this study suggest that comprehensive strategies, considering both structural factors and individual characteristics as well as differentiated wellbeing profiles, could be useful in promoting more balanced academic environments.

These results should be interpreted in light of several limitations. The low overall response rate (6.8%; *n* = 742) and its marked variability across groups (students: 4.2%; ARS: 12.9%; TASS: 31.6%) limit sample representativeness and suggest a potential non-response bias. The underrepresentation of students may reflect self-selection of more engaged profiles, although the direction and magnitude of this bias cannot be determined without a formal non-response analysis. Nevertheless, the stability of the factor solutions observed through bootstrapping with 500 replications supports the internal robustness of the analyses conducted. Consequently, the results can be considered analytically robust, although provisional in terms of external validity. Future studies should replicate these findings using stratified probability sampling designs to strengthen their generalisability to the wider university community.

The cross-sectional design precludes establishing causal relationships among the variables analyzed. In addition, restricting the study to a single institution (UVic-UCC) limits the generalizability of the findings to other university contexts, both geographically and socioeconomically. The attrition observed during questionnaire completion may have introduced attrition bias, potentially altering the profile of the final respondents. Although the applied exclusion criteria (minimum response times) reinforced data quality, they may also have intensified self-selection bias toward more motivated participants. As a counterbalance, the study is distinguished by the application of a combined exploratory analytical approach (LPA + Logistic Regression) and by achieving adequate sample sizes for the planned intragroup analyses, while acknowledging that external validation would be necessary before the findings can be extended beyond the study context.

As noted in Section 2.5, the retained one-factor solution should be interpreted as an operational and parsimonious approximation of global psychological wellbeing rather than as a definitive measurement model. Although factor loadings and reliability indices supported its use for the exploratory profile analysis, the elevated RMSEA and RMSR above Kelley's criterion indicate residual model misfit. Future studies should replicate these findings in independent samples and examine bifactor or hierarchical models to achieve a more comprehensive representation of the construct's latent structure. Because the wellbeing profiles reported throughout this study rest on this operational index, they should be interpreted as conditional on an index with imperfect model fit. As a sensitivity check, we note that the Latent Profile Analysis (Section 2.5, Table 8) was estimated directly on the five continuous indicators rather than on a collapsed one-factor score, indicating that the two-cluster solution does not mechanically depend on the fit of the one-factor CFA; nonetheless, replication with alternative measurement specifications remains warranted.

An additional limitation of the group-specific clustering approach is that the resulting low- and high-wellbeing profiles reflect the internal distribution of each subpopulation and are not directly equivalent across groups in a strict quantitative sense. Because the aim of the study was exploratory and intragroup, identifying wellbeing typologies within each collective to inform tailored interventions, this design choice was theoretically justified; however, it precludes formal cross-group profile comparisons. Future studies should replicate these findings using stratified probability sampling designs to strengthen their generalizability and apply multigroup LPA with measurement invariance testing to establish the extent to which wellbeing profiles are structurally homologous across students, ARS, and TASS, thereby enabling more rigorous between-group comparisons. Such analyses should also consider broader psychometric validity, interpretive equivalence, and contextual norming requirements when assessing psychological constructs across distinct subpopulations (Dergaa et al., 2026).

Another possible limitation would correspond to the absence of an a priori statistical power analysis, meaning that non-significant findings, particularly in the ARS and TASS subgroups (*n* ≈ 180), may reflect a Type II error rather than a true absence of association. Similarly, the very low frequency of drug use in the student sample (*n* = 7) required the use of Firth's penalized regression, and the resulting estimate should therefore be interpreted with caution. Larger samples are needed to draw firm conclusions regarding low-prevalence predictors.

### 4.5 Future directions

The integration of latent profile analysis and logistic regression provides an exploratory methodological framework for characterizing wellbeing heterogeneity within the university community. Replication in independent samples and external validation would be needed before these findings could inform risk screening or the prioritization of evidence-based interventions in other higher education institutions.

The findings of this study support the need for longitudinal, multi-cohort research to evaluate the temporal stability of wellbeing profiles (low vs. high) and the rates of transition between them, with the aim of identifying critical windows for intervention throughout the academic cycle.

Additionally, multi-institutional and transnational replication is required, including public and private universities and diverse socioeconomic strata, combined with factorial invariance analyses to validate the generalizability of the profiles and explore contextual moderators such as organizational culture and budget allocation.

The use of longitudinal Vector Autoregressive (VAR) or Graphical Vector Autoregressive (GVAR) models would allow the examination of temporal interdependencies and feedback dynamics among different dimensions of psychological wellbeing.

Finally, a promising methodological development could involve integrating latent profiles with prospectively validated administrative and psychosocial data to develop exploratory decision-support tools, pending rigorous external validation, ethical evaluation, and prospective testing.

## 5 Conclusions

This study, using latent profile analysis (LPA) and multivariate analysis, suggests the presence of two differentiated profiles of psychological wellbeing within each subgroup of the university community. Within-group analyses revealed a higher within-group prevalence of the low wellbeing profile among TASS (70.3%) and students (64.1%), and a more balanced distribution among ARS (49.7%); however, given that profiles were estimated independently within each group, these figures reflect intragroup distributions and should not be read as direct between-group contrasts. Consistent with this pattern, the results indicate that economic status is a consistent structural predictor across the three groups, whereas perceived health, although significantly associated with profile membership among Students and TASS, did not reach significance among ARS (OR = 0.70, *p* = 0.234); other variables such as reversed cynicism (R-CY) and psychological distress (R-GSI) exhibit differential weighting depending on the group.

Taken together, these exploratory findings suggest that psychological wellbeing may vary across groups within the university community and that future research could examine whether differentiated institutional responses are warranted. The integration of latent profile analysis and logistic regression provides a preliminary framework for characterizing wellbeing heterogeneity and exploring sociodemographic and health-related factors associated with profile membership. However, given the single-institution setting, the cross-sectional design, and the 6.8% self-selected response rate, the findings should not be interpreted as supporting specific screening, resource-allocation, or intervention-planning decisions. Although the analytical methods produced broadly consistent patterns, independent replication, external validation, and studies using stratified probability sampling are required before any organizational, academic, emotional, or financial interventions can be recommended.

## Figures and Tables

**Figure:**
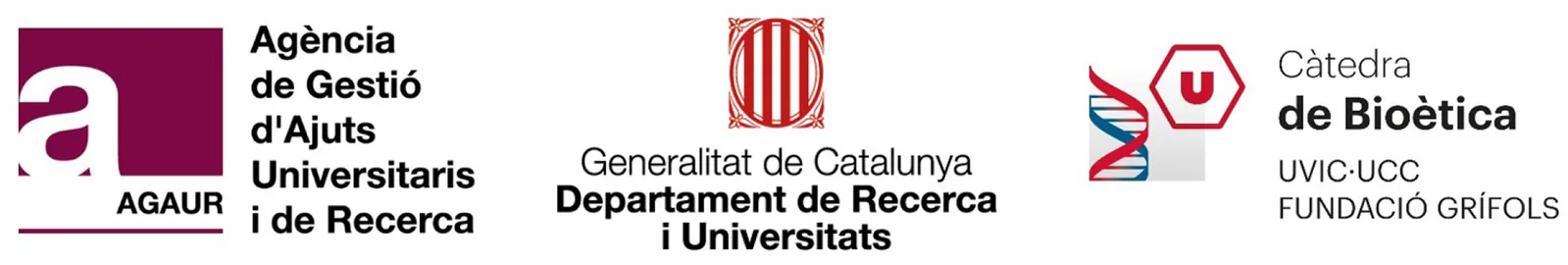


## Data Availability

The raw data supporting the conclusions of this article will be made available by the authors, without undue reservation.

## References

[B1] Akbas T. T. Durak I. Cetin A. Karkin N. (2018). Cynicism as mediating variable between leadership support and emotional burnout: administrative support staff in turkish universities. Transylv. Rev. Admin. Sci. 53E, 5–21. doi: 10.24193/tras.53E.1

[B2] Andreu Y. Galdón M. J. Ferrando M. Cañeva P. Ibañez E. Dóminguez M. L. (2008). Obtaining evidence of validity for the Spanish version of the Brief Symptom Inventory (BSI). Span. J. Psychol. 11, 626–636. doi: 10.1017/S113874160000463718988448

[B3] Bakker A. B. Demerouti E. (2007). The job demands-resources model: state of the art. J. Manag. Psychol. 22, 309–328. doi: 10.1108/02683940710733115

[B4] Bakker A. B. Demerouti E. (2017). Job demands-resources theory: taking stock and looking forward. J. Occup. Health Psychol. 22, 273–285. doi: 10.1037/ocp0000056 27732008

[B5] Baruch Y. Holtom B. C. (2008). Survey response rate levels and trends in organizational research. Hum. Relat. 61, 1139–1160. doi: 10.1177/0018726708094863

[B6] Beck D. Lenhardt U. (2019). Consideration of psychosocial factors in workplace risk assessments: Findings from a company survey in Germany. Int. Arch. Occup. Environ. Health 92, 435–451. doi: 10.1007/s00420-019-01416-5 30756179 PMC6420464

[B7] Bourdieu P. (1988). Homo Academicus. Stanford, CA: Stanford University Press.

[B8] Bruno A. Buono C. Falco A. Brondino M. Capone V. Dell'Aversana G. . (2024). First validation of the technical and administrative staff quality of life at work tool (TASQ@work) in academia. Front. Psychol. 15, 1–19. doi: 10.3389/fpsyg.2024.1346556 38680287 PMC11048465

[B9] Cadena-Povea H. Hernández-Martínez M. Bastidas-Amador G. Torres-Andrade H. (2025). What pushes university professors to burnout? A systematic review of sociodemographic and psychosocial determinants. Int. J. Environ. Res. Public Health 22:1214. doi: 10.3390/ijerph22081214 40869800 PMC12386177

[B10] Chalghaf N. Chokri I. Dhahbi W. Ceylan H. Bragazzi N. L. Muntean R. I. . (2026). Burnout across healthcare, educational, and professional populations: a comprehensive scoping review. J. Multidiscip. Healthc. 19, 1–17. doi: 10.2147/JMDH.S564113 41877964 PMC13007544

[B11] Chirico F. Heponiemi T. Pavlova M. Zaffina S. Magnavita N. (2019). Psychosocial risk prevention in a global occupational health perspective. A descriptive analysis. Int. J. Environ. Res. Public Health 16:2470. doi: 10.3390/ijerph16142470 31336707 PMC6678173

[B12] Coleman A. Ali A. (2025). Emotional intelligence: its importance to HE professional services team members during challenging times. Manag. Educ. 39, 59–65. doi: 10.1177/08920206221085794

[B13] Corpuz J. C. G. (2023). Enhancing wellbeing in the university workplace: the context of higher educational institution. J. Prim. Care Commun. Health 14:21501319231181488. doi: 10.1177/21501319231181488 37326366 PMC10278421

[B14] De Beer L. T. van der Vaart L. Escaffi-Schwarz M. De Witte H. Schaufeli W. B. (2024). Maslach Burnout Inventory-General Survey: a systematic review and meta-analysis of measurement properties. Eur. J. Psychol. Assess. 40, 360–375. doi: 10.1027/1015-5759/a000797

[B15] Departament de Recerca i Universitats (2023). Estudi sobre la salut mental en el sistema universitari de Catalunya. Available online at: https://hdl.handle.net/20.500.14344/461 (Accessed September 11, 2025).

[B16] Departament d'Empresa i Treball (2021). Estratégia Catalana de Seguretat i Salut Laboral 2021–2026. Generalitat de Catalunya. Available online at: https://treball.gencat.cat/ca/consell_relacions_laborals/ci/Estrategia-Catalana-de-Seguretat-i-Salut-Laboral-2021–2026/

[B17] Dergaa I. Chalghaf N. Dhahbi W. Irandoust K. Ceylan H. Stefanica V. . (2026). Measuring minds across generations: A theoretical framework on psychometric validity, interpretive frameworks, and contextual norming in cross-cohort psychological research. Front. Psychol. 17:1794790. doi: 10.3389/fpsyg.2026.1794790 42495233 PMC13393458

[B18] Derogatis L. R. (2013). Brief Symptom Inventory 18 (BSI-18): Administration, Scoring and Procedures Manual. Minneapolis, MN: NCS Pearson.

[B19] Díaz-Patiño D. G. Anaya-Velasco A. (2022). Relación de los factores de riesgo psicosocial y salud en trabajadores de universidades: revisión sistemática. Rev. Iberoamer. Educación Superior 13, 182–203. doi: 10.22201/iisue.20072872e.2022.38.1518

[B20] Diener E. Emmons R. A. Larsen R. J. Griffin S. (1985). The satisfaction with life scale. J. Pers. Assess. 49, 71–75. doi: 10.1207/s15327752jpa4901_13 16367493

[B21] Gil-Monte P. R. (2002). Validez factorial de la adaptación al español del Maslach Burnout Inventory-General Survey (MBI-GS). Salud Pública de México 44, 33–40. doi: 10.1590/S0036-36342002000100005 11910717

[B22] Guelmami N. Saidane M. Ben Aissa M. Rebhi M. Chalghaf N. Azaiez F. . (2023). Multidimensional assessment of student mental health during the COVID-19 pandemic: a latent profile analysis integrating positive psychology. New Asian J. Med. 1, 20–29. doi: 10.61186/najm.1.1.20

[B23] Guevara Bedoya L. M. Ocampo Agudelo N. (2014). Propiedades psicométricas de confiabilidad y validez del Maslach Burnout Inventory-General Survey (MBI-GS). Rev. Interam. Psicol. Organ. 33, 128–142. doi: 10.21772/ripo.v33n2a04

[B24] Guinez-Perez M. Araya-Guzman S. Salazar-Concha C. (2022). “Exploring factors that affect technological anxiety (technoanxiety) of university administrative staff,” in Proceedings of the 17th Iberian conference on information systems and technologies (CISTI) (IEEE). doi: 10.23919/CISTI54924.2022.9820599

[B25] Hair J. F. Anderson R. E. Tatham R. L. Black W. C. (1999). Multivariate Data Analysis. Upper Saddle River, NJ: Prentice Hall 5 edition.

[B26] Hair J. F. Black W. C. Babin B. J. Anderson R. E. (2019). Multivariate Data Analysis. Hampshire, UK: Cengage, 8 edition.

[B27] Hederich-Martínez C. Caballero-Domínguez J. F. (2016). Validación del Maslach Burnout Inventory-Student Survey (MBI-SS) en estudiantes universitarios colombianos. Rev. CES Psicol. 9, 1–15. doi: 10.21615/cesp.9.1.1

[B28] Hernández-Torrano D. Ibrayeva L. (2025). Who feels good at university? Exploring the prevalence, profiles, and determinants of mental health in higher education students using a person-centered approach. Asia-Pacific Educ. Resear. 34, 93–107. doi: 10.1007/s40299-024-00839-0

[B29] Innstrand S. T. Christensen M. (2018). Healthy universities. The development and implementation of a holistic health promotion intervention programme especially adapted for staff working in the higher educational sector: the ARK study. Global Health Promot. 27, 68–76. doi: 10.1177/1757975918786877 30328755

[B30] Jayman M. Glazzard J. Rose A. (2022). Tipping point: the staff wellbeing crisis in higher education. Front. Educ. 7:929335. doi: 10.3389/feduc.2022.929335

[B31] Jelleli H. Ben Aissa M. Kaddech N. Saidane M. Guelmami N. Bragazzi N. L. . (2024). Examining the interplay between physical activity, problematic internet use and the negative emotional state of depression, anxiety and stress: insights from a moderated mediation path model in university students. BMC Psychol. 12:406. doi: 10.1186/s40359-024-01736-3 39054507 PMC11274773

[B32] Kelloway E. K. Dimoff J. K. Gilbert S. (2023). Mental health in the workplace. Annu. Rev. Organ. Psychol. Organ. Behav. 10, 363–387. doi: 10.1146/annurev-orgpsych-120920-050527

[B33] Keyes C. L. M. (2002). The mental health continuum: from languishing to flourishing in life. J. Health Soc. Behav. 43, 207–222. doi: 10.2307/3090197 12096700

[B34] Keyes C. L. M. (2007). Promoting and protecting mental health as flourishing: a complementary strategy for improving national mental health. Am. Psychol. 62, 95–108. doi: 10.1037/0003-066X.62.2.95 17324035

[B35] Kinman G. Wray S. (2013). Higher stress: a survey of stress and wellbeing among staff in higher education. Technical report, University and College Union.

[B36] Kirsten W. (2022). The evolution from occupational health to healthy workplaces. Am. J. Lifestyle Med. 18, 64–74. doi: 10.1177/15598276221113509 39184266 PMC11339767

[B37] Koren H. Milaković M. Bubaš M. Bekavac P. Bekavac B. Bucić L. . (2023). Psychosocial risks emerged from COVID-19 pandemic and workers' mental health. Front. Psychol. 14:1148634. doi: 10.3389/fpsyg.2023.1148634 37303891 PMC10254389

[B38] Kritikou M. Giovazolias T. (2022). Emotion regulation, academic buoyancy, and academic adjustment of university students within a self-determination theory framework: a systematic review. Front. Psychol. 13:1057697. doi: 10.3389/fpsyg.2022.1057697 36524164 PMC9746693

[B39] Lee J. J. Stensaker B. (2021). Research on internationalisation and globalisation in higher education–Reflections on historical paths, current perspectives and future possibilities. Eur. J. Educ. 56, 157–168. doi: 10.1111/ejed.12448

[B40] Li W. Zhao Z. Chen D. Peng Y. Lu Z. (2022). Prevalence and associated factors of depression and anxiety symptoms among college students: a systematic review and meta-analysis. J. Child Psychol. Psychiat. 63, 1222–1230. doi: 10.1111/jcpp.13606 35297041

[B41] Lindholm M. Reiman A. Tappura S. (2024). The evolution of new and emerging occupational health and safety risks: a qualitative review. Work 79, 1–19. doi: 10.3233/WOR-230005 38701168 PMC11491999

[B42] Little R. J. A. Rubin D. B. (2002). Statistical Analysis with Missing Data. Hoboken, NJ: Wiley, 2 edition. doi: 10.1002/9781119013563

[B43] Lorenzo-Seva U. (2026). Mild-restricted confirmatory factor analysis: a new approach to assessing population factor models. Commun. Stat. Simul. Comput. 55, 1656–1674. doi: 10.1080/03610918.2025.2453473

[B44] Lubke G. H. Muthén B. (2005). Investigating population heterogeneity with factor mixture models. Psychol. Methods 10, 21–39. doi: 10.1037/1082-989X.10.1.21 15810867

[B45] Madigan D. J. Curran T. (2021). Does burnout affect academic achievement? A meta-analysis. Educ. Psychol. Rev. 33, 387–405. doi: 10.1007/s10648-020-09533-1

[B46] March-Amengual J. M. Cambra Badii I. Casas-Baroy J. C. Altarriba C. Comella Company A. Pujol-Farriols R. . (2022). Psychological distress, burnout, and academic performance in first year college students. Int. J. Environ. Res. Public Health 19:3356. doi: 10.3390/ijerph19063356 35329044 PMC8953100

[B47] Marginson S. (2008). Global field and global imagining: bourdieu and worldwide higher education. Br. J. Sociol. Educ. 29, 303–315. doi: 10.1080/01425690801966386

[B48] Masyn K. E. (2013). “Latent class analysis and finite mixture modeling,” in The Oxford Handbook of Quantitative Methods, ed. T. D. Little (Oxford: Oxford University Press), 551–611. doi: 10.1093/oxfordhb/9780199934898.013.0025

[B49] Meeks K. Peak A. S. Dreihaus A. (2023). Depression, anxiety, and stress among students, faculty, and staff. J. Am. College Health 71, 348–354. doi: 10.1080/07448481.2021.1891913 33759719

[B50] Mooi E. Sarstedt M. (2011). A Concise Guide to Market Research. Berlin: Springer. doi: 10.1007/978-3-642-12541-6

[B51] Moreno A. (2022). La desconexión digital en el teletrabajo como medida de prevención de los riesgos psicosociales. Anuario IET de Trabajo y Relaciones Laborales 8, 111–138. doi: 10.5565/rev/aiet.109

[B52] Mudrak J. Zabrodska K. Kveton P. Jelinek M. Blatny M. Solcova I. . (2018). Occupational wellbeing among university faculty: a job demands-resources model. Res. High. Educ. 59, 325–348. doi: 10.1007/s11162-017-9467-x

[B53] Nylund K. L. Asparouhov T. Muthén B. O. (2007). Deciding on the number of classes in latent class analysis and growth mixture modeling: a Monte Carlo simulation study. Struct. Equat. Model. 14, 535–569. doi: 10.1080/10705510701575396

[B54] Odriozola-González P. Planchuelo-Gómez Á. Irurtia M. J. de Luis-García R. (2020). Psychological effects of the covid-19 outbreak and lockdown among students and workers of a spanish university. Psychiatry Res. 290:113108. doi: 10.1016/j.psychres.2020.113108 32450409 PMC7236679

[B55] Pereira P. C. Mour ao M. F. Carvalho P. M. (2023). Influence of psychosocial risks on the quality of life of employees in a higher education institution: a critical perspective. Int. J. Bus. Manag. Stud. 4, 26–34. doi: 10.56734/ijbms.v4n11a4

[B56] Saidane M. Guelmami N. Dhahbi W. Ayachi F. Ceylan H. . . (2026). Psychological distress, digital behavioral risks, and physical activity as predictors of subjective wellbeing: a machine learning study in a large sample of community-dwelling adults. Front. Behav. Neurosci. 20:1876257. doi: 10.3389/fnbeh.2026.1876257 42488360 PMC13388298

[B57] Salanova M. Schaufeli W. B. Llorens S. Peiró J. M. Grau R. (1999). Maslach Burnout Inventory-General Survey; Spanish version (MBI-GS, 15 ítems). PsycTESTS. Database record. doi: 10.1037/t05260-000

[B58] Schaufeli W. B. (2003). “Past performance and future perspectives of burnout research,” in Handbook of Work and Health Psychology, eds. M. J. Schabracq, J. A. M. Winnubst, and C. L. Cooper (Chichester, UK: Wiley), 383–429.

[B59] Schaufeli W. B. Martínez I. M. Pinto A. M. Salanova M. Bakker A. B. (2002). Burnout and engagement in university students: a cross-national study. J. Cross Cult. Psychol. 33, 464–481. doi: 10.1177/0022022102033005003

[B60] Standing G. (2011). The Precariat: The New Dangerous Class. London: Bloomsbury Academic. doi: 10.5040/9781849664554

[B61] Torales J. Torres-Romero A. Barrios I. O'Higgins M. Caycho-Rodríguez T. Castaldelli-Maia J. M. . (2025). Mental health, emotional regulation, and psychosocial work factors among scientific researchers: a cross-sectional study from Paraguay. Brain Sci. 15, 1–16. doi: 10.3390/brainsci15010065 39851432 PMC11763664

[B62] Trabelsi K. Ammar A. Masmoudi L. Boukhris O. Chtourou H. Bouaziz B. . (2021). Sleep quality and physical activity as predictors of mental wellbeing variance in older adults during COVID-19 lockdown: ECLB COVID-19 international online survey. Int. J. Environ. Res. Public Health 18:4329. doi: 10.3390/ijerph18084329 33921852 PMC8073845

[B63] Universitat de Vic-Universitat Central de Catalunya (2025). Memória institucional 2024–2025. Available online at: https://hdl.handle.net/10854/180501 (Accessed June 10, 2026).

[B64] Vázquez C. Duque A. Hervás G. (2013). Satisfaction with Life Scale in a representative sample of Spanish adults: validation and normative data. Spanish J. Psychol. 16:e82. doi: 10.1017/sjp.2013.82 24230945

[B65] World Health Organization (2022a). COVID-19 pandemic triggers 25% *increase in prevalence of anxiety and depression worldwide*. Available online at: https://www.who.int/news/item/02–03-2022-covid-19-pandemic-triggers-25-increase-in-prevalence-of-anxiety-and-depression-worldwide

[B66] World Health Organization (2022b). Mental health at work: policy brief . Available online at: https://iris.who.int/bitstream/handle/10665/362983/9789240057944-eng.pdf

[B67] World Health Organization (2022c). World Mental Health Report: Transforming Mental Health for All. Geneva: World Health Organization

[B68] Worsley J. D. Pennington A. Corcoran R. (2022). Supporting mental health and wellbeing of university and college students: a systematic review of review-level evidence of interventions. PLoS ONE 17:e0266725. doi: 10.1371/journal.pone.0266725 35905058 PMC9337666

[B69] Wray S. Kinman G. (2021). Supporting staff wellbeing in higher education. Education Support. Available online at: https://www.educationsupport.org.uk/media/x4jdvxpl/es-supporting-staff-wellbeing-in-he-report.pdf

