# Supplementary material for: Heterogeneity in psychological wellbeing within the university community: a latent profile analysis of wellbeing profiles and associated factors
Source: Front Psychol. 2026 Aug 12;17:1815021. doi: 10.3389/fpsyg.2026.1815021 (PMC13509032; doi:10.3389/fpsyg.2026.1815021)
Supplement: Supplementary file 1 [file Data_Sheet_1.pdf]

1 SUPPLEMENTARY TABLES AND FIGURES

1.1 Tables

Table S1. Performance metrics of the Random Forest model across all groups.

Group	AUC	Accuracy	Recall	Precision
Students	0.996 ± 0.004	0.962 ± 0.018	0.925 ± 0.040	0.969 ± 0.028
ARS	0.996 ± 0.007	0.947 ± 0.047	0.958 ± 0.052	0.944 ± 0.072
TASS	0.986 ± 0.008	0.924 ± 0.043	0.855 ± 0.093	0.903 ± 0.104

0.8

Note: AUC = Area Under the ROC Curve; Accuracy = proportion of correctly classified cases; Recall = sensitivity (true positive rate); Precision = positive predictive value. Values are reported as mean ± SD across 5-fold stratified cross-validation (Random Forest classifier, 50 decision trees, random_state = 42). This model was trained and evaluated on the same five variables used to derive the well-being clusters; the resulting near-perfect values reflect this circularity by construction and do not constitute external validation of the cluster solution. See Section 3.1 of the main manuscript for full interpretation.

Note: This model was trained and evaluated on the same five variables used to derive the well-being clusters; the resulting near-perfect AUC values reflect this circularity by construction and do not constitute external validation of the cluster solution. See Section 3.1 of the main manuscript for full interpretation.
